# Combined Scutellarin and C_18_H_17_NO_6_ Imperils the Survival of Glioma: Partly Associated With the Repression of PSEN1/PI3K-AKT Signaling Axis

**DOI:** 10.3389/fonc.2021.663262

**Published:** 2021-09-09

**Authors:** Xiu-Ying He, Yang Xu, Qing-Jie Xia, Xiao-Ming Zhao, Shan Li, Xiao-Qiong He, Ru-Rong Wang, Ting-Hua Wang

**Affiliations:** ^1^ Institute of Neurological Disease, Department of Anesthesiology, Translational Neuroscience Center, West China Hospital, Sichuan University, Chengdu, China; ^2^ Institute of Neuroscience, Laboratory Zoology Department, Kunming Medical University, Kunming, China; ^3^ School of Public Health, Kunming Medical University, Kunming, China

**Keywords:** C_18_H_17_NO_6_, PSEN1, glioma, PI3K-Akt signaling, scutellarin

## Abstract

Glioma, the most common intracranial tumor, harbors great harm. Since the treatment for it has reached the bottleneck stage, the development of new drugs becomes a trend. Therefore, we focus on the effect of scutellarin (SCU) and its combination with C_18_H_17_NO_6_ (abbreviated as *combination*) on glioma and its possible mechanism in this study. Firstly, SCU and C_18_H_17_NO_6_ both suppressed the proliferation of U251 and LN229 cells in a dose-dependent manner, and C_18_H_17_NO_6_ augmented the inhibition effect of SCU on U251 and LN229 cells *in vitro*. Moreover, there was an interactive effect between them. Secondly, SCU and C_18_H_17_NO_6_ decreased U251 cells in G2 phase and LN229 cells in G2 and S phases but increased U251 cells in S phase, respectively. Meanwhile, the combination could further reduce U251 cells in G2 phase and LN229 cells in G2 and S phases. Thirdly, SCU and C_18_H_17_NO_6_ both induced the apoptosis of U251 and LN229. The combination further increased the apoptosis rate of both cells compared with the two drugs alone. Furthermore, SCU and C_18_H_17_NO_6_ both inhibited the lateral and vertical migration of both cells, which was further repressed by the combination. More importantly, the effect of SCU and the combination was better than positive control-temozolomide, and the toxicity was low. Additionally, SCU and C_18_H_17_NO_6_ could suppress the growth of glioma *in vivo*, and the effect of the combination was better. Finally, SCU and the combination upregulated the presenilin 1 (PSEN1) level but inactivated the phosphatidylinositol 3−kinase (PI3K)-protein kinase B (AKT) signaling *in vitro* and *in vivo*. Accordingly, we concluded that scutellarin and its combination with C_18_H_17_NO_6_ suppressed the proliferation/growth and migration and induced the apoptosis of glioma, in which the mechanism might be associated with the repression of PSEN1/PI3K-AKT signaling axis.

## Introduction

Glioma is the most common intracranial tumor, accounting for 80% of all primary malignant central nervous system tumors (1–3), which harbors all the characteristics of malignant tumors, including proliferation, invasion, and metastasis. At present, surgery, radiotherapy, chemotherapy, immunotherapy, X-knife, and gamma knife are common treatments for glioma. Although great achievements have been made, the efficacy is still not satisfactory, which leads to the recurrence of gliomas mostly within 1–5 years and imposes a great burden on patients and society (4, 5). As you know, the treatment of glioma has entered a bottleneck stage, and the development and application of new drugs becomes a trend.

Scutellarin, one of the main effective monomers of *Erigeron breviscapus*, belongs to the active ingredient of flavonoids (6–8). Its pharmacological effects are extensive, such as, expansion of arterioles, reduction of peripheral resistance, and antibrain and myocardial ischemia (6, 9). Among all the effects, it is worth pondering that scutellarin possesses the antitumor effect in most tumors. It was reported that scutellarin, with a strong PKM2 activation effect, could serve as an anticancer drug to suppress cell growth in prostate cancer (10–12). Moreover, the conjugated drugs of scutellarin, such as scutellarin polyrotaxane (SCU-PR) and scutellarin-cyclodextrin conjugate, could elevate antitumor activity (13, 14). In addition, the functional mechanism of scutellarin is clear. In colorectal cancer, scutellarin took effect through the following mechanisms: (1) to reduce cell viability and sensitize RSV- and 5-FU-triggered apoptosis by modulating the expression of p53 and Bcl2/Bax (7, 15); (2) to induce cell death by participating in metabolism and regulating the factors related with cell cycle and transcription (16). In liver cancer, scutellarin inhibited cell proliferation and the metastasis and invasion into the lung and liver by downregulating STAT3/Girdin/Akt signaling (17, 18). Although scutellarin exerts anticancer effects through different mechanisms in various tumors, its role in gliomas is still poorly studied.

Additionally, as a newly developed antitumor drug, C_18_H_17_NO_6_ harbors a strong antitumor effect *in vitro* and *in vivo* with low toxicity (Patent No.: 201710388136.8). *In vitro* experiments have shown that C_18_H_17_NO_6_ exerted stronger inhibitory effect on lung cancer, liver cancer, bladder cancer, breast cancer, and nasopharyngeal carcinoma cell lines than cisplatin and 5-fluorouracil and was superior to paclitaxel (Patent No.: 201710388136.8). Moreover, C_18_H_17_NO_6_ significantly restrained the growth of liver cancer *in vivo* (Patent No.: 201710388136.8). However, the effect of it on glioma is still not well understood.

Presenilin 1 (PSEN1) is an important component of γ-secretase, and its mutations would lead to apoptosis, neurodegeneration, and cognitive decline, which might cause the occurrence and progression of Alzheimer’s disease (19, 20). Somavarapu et al. found that there were nearly 200 mutations in the *PSEN1* gene, which altered amyloid precursor metabolism or increased neuronal apoptosis, resulting in early Alzheimer’s disease (21, 22). Moreover, wild-type PSEN1 expression was elevated in neural regeneration and differentiation (23). It could be seen that PSEN1 played a key role in promoting apoptosis and differentiation. What is more, recent studies have found that PSEN1 had great influence on tumor progression (24) and drug resistance (25).

Since PSEN1 inhibited cell proliferation and induced apoptosis by suppressing phosphatidylinositol 3−kinase/protein kinase B (PI3K/AKT) signaling (26, 27), we further explored whether PI3K/AKT signaling was involved in the inhibitory effect on glioma by scutellarin and C_18_H_17_NO_6_. The genes of PI3K/AKT pathway were the most frequently altered in human cancers, and abnormal activation and molecular alterations in this pathway were associated with tumorigenesis, cell transformation, tumor progression, and drug resistance (28). In phosphatase and tensin homolog (PTEN)-deficient prostate cancer, PTEN deletion resulted in AKT activation, thereby driving prostate cancer metastasis (29). Moreover, PI3K/AKT signaling was involved in the enrichment of nuclear upstream element binding protein (FBP)1 and FBP2 in hepatocellular carcinoma cells, which were significantly correlated with the expression of the proliferation marker Ki67 (30). Furthermore, molecular changes in PI3K/AKT/mTOR pathway, containing mutations, copy number, protein, or RNA, have been detected in 11,219 human cancers (32 major cancer types included) (31). Thus, the drugs targeting PI3K/AKT signaling for treating patients with solid tumors and hematological malignancies would be the focus (28).

Based on the above, we proposed a hypothesis that scutellarin and its combination with C_18_H_17_NO_6_ can suppress the progression of glioma by repressing of PSEN1/PI3K-AKT signaling axis. Therefore, the purpose of this study was to investigate the role of scutellarin and C_18_H_17_NO_6_ in glioma and the relative mechanism.

## Results

### Scutellarin and C_18_H_17_NO_6_ Suppress the Proliferation and Migration of Glioma Cells

**Figure 1:**
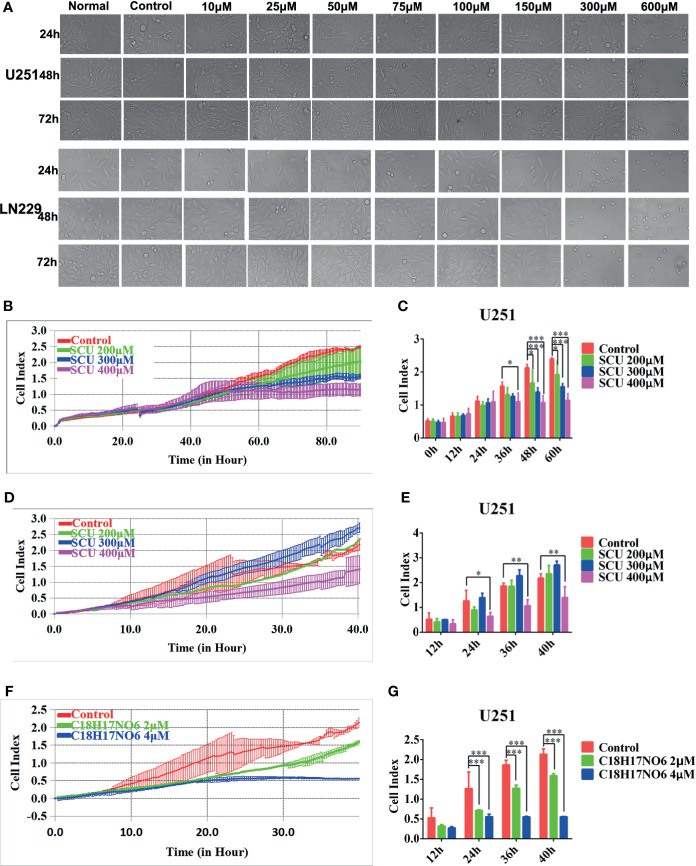
Scutellarin and C_18_H_17_NO_6_ suppress the proliferation and migration of glioma cells. **(A)** The morphological change of U251 and LN229 cells were observed at 24, 48, and 72 h after the intervention of SCU at different concentrations. Normal: administrated with complete medium; control: 0.2% dimethyl sulfoxide (DMSO, Sigma). **(B, C)** SCU suppressed the proliferation of U251 cells. **(B)** Cell index was recorded at 15 min interval after inoculation. **(C)** Cell index was recorded after intervention by SCU for 0, 12, 24, 36, 48, and 60 (h) **(D, E)** SCU suppressed the migration of U251 cells. Here, the inoculated U251 cells were intervened with 0.133% DMSO (control group), SCU 200 μM, SCU 300 μM, and SCU 400 μM for 48 h in advance. **(D)** Cell index was recorded at 15 min interval. **(E)** Cell index at 12, 24, 36, and 40 h of inoculation. **(F, G)** C_18_H_17_NO_6_ also controlled the migration of U251 cells. The inoculated U251 cells were intervened with 0.04% DMSO (control group), C_18_H_17_NO_6_ 2 μM, and C_18_H_17_NO_6_ 4 μM for 48 h beforehand. **(F)** Cell index was recorded at 15 min interval. **(G)** Cell index at 12, 24, 36, and 40 h of inoculation. The data were presented as mean ± standard deviation (SD) (*n* = 3). Compared with the control group: *p < 0.05, **p < 0.01, and ***p < 0.001.

Consistent with the results reported previously (32), the inhibitory effect on glioma cells U251 and LN229 was also enhanced with the increase of SCU concentration. As shown in **Figure 1A**, the number of U251 and LN229 living cells was reduced, especially when the SCU dose reached 300 μM or more. Moreover, the IC_50_ (the concentration when the inhibitory efficiency reaches 50%) of SCU in U251 and LN229 cells was 267.4 and 286.1 μM, respectively (32). Therefore, the dose range of SCU for subsequent study was set to 100~400 μM. In addition, the effect of SCU on the proliferation and migration of U251 cells was also continuously monitored by xCELLigence Real Time Cell Analyzer (RTCA, ACEA, San Diego, CA, USA). On the one hand, the cell index curves of proliferation in all groups basically coincided before drug administration, but after administration, the cell index of U251 decreased as the SCU dose increased (**Figures 1B, C** **)**. At 36 h of intervention, compared with the control group (0.133% dimethyl sulfoxide (DMSO)), the cell index of all SCU groups with different concentrations decreased, but only the difference between SCU 400 μM and the control group was significant (*p* < 0.05) (**Figures 1B, C** **)**. At 48 and 60 h, the cell index of SCU 200, 300, and 400 μM groups declined significantly in comparison with the control group (*p* < 0.05) (**Figures 1B, C** **)**. On the other hand, we found that compared with the control group (0.133% DMSO), the cell index of migration decreased when the concentration of SCU was 400 μM, and the difference was all statistically significant at 24, 36, and 40 h of intervention (*p* < 0.05) (**Figures 1D, E** **)**.

Additionally, the IC_50_ of C_18_H_17_NO_6_ in U251 and LN229 cells was 3.926 and 7.345 μM, respectively, so the concentration range of C_18_H_17_NO_6_ in this study was set to 1~5 μM. To further determine the efficacy of C_18_H_17_NO_6_, the U251 cells, intervened by 0.04% DMSO (control group), C_18_H_17_NO_6_ 2 μM, and C_18_H_17_NO_6_ 4 μM for 48 h, were collected and inoculated into CIM plates 16, and the migration was also monitored by xCELLigence Real Time Cell Analyzer (ACEA). The results showed that compared with the control group, the cell index was reduced with the increase of the C_18_H_17_NO_6_ concentration (**Figures 1F, G** **)**. Moreover, at 24, 36, and 40 h, the differences between the C_18_H_17_NO_6_ 2 μM and control group and between the C_18_H_17_NO_6_ 4 μM and control group were statistically significant (*p* < 0.05), and the cell index in the C_18_H_17_NO_6_ 4 μM group was lower than that in the C_18_H_17_NO_6_ 2 μM group (**Figures 1F, G** **)**.

### The Toxicity of Scutellarin and C_18_H_17_NO_6_ on Astrocytes and Neurons

**Figure 2:**
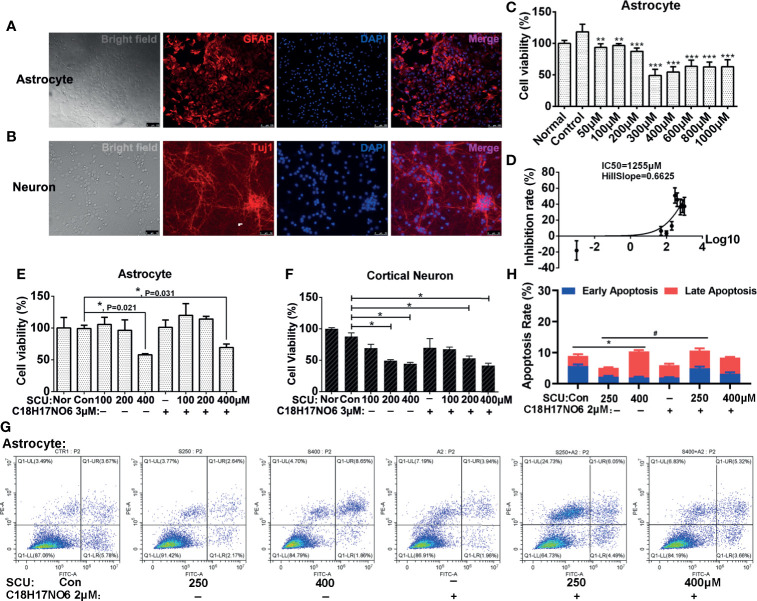
The toxicity of scutellarin and C1_8_H_17_NO_6_ on astrocytes and neurons. **(A, B)** Purity identification of P2 generation astrocytes and P1 generation cortical neurons cultured for 6 days. **(C)** The effect of SCU on cell viability of astrocytes. **(D)** The IC_50_ curve of SCU in astrocytes. **(E, F)** The effect of SCU and its combination with C_18_H_17_NO_6_ 3 μM on cell viability of astrocytes and cortical neurons, respectively. **(G, H)** The effect of SCU and its combination with C_18_H_17_NO_6_ 2 μM on the apoptosis of astrocytes. GFAP, astrocyte marker; Tuj1, neuron marker; Nor, normal cells without intervention; Con, control group; cells were treated with 0.163% DMSO. The data were shown as mean ± standard deviation (*n* = 3). Compared with the control group: *p < 0.05, **p < 0.01, and ***p < 0.001; SCU x vs. SCU x + C18H17NO6 2 μM: ^#^P < 0.05.

To investigate the toxic effect of SCU and C_18_H_17_NO_6_ on normal cells, astrocytes and cortical neurons from neonatal SD rats were isolated, cultured, and purified. The cells included in the toxicity analysis were P2 generation astrocytes (**Figure 2A**) and P1 generation cortical neurons cultured for 6 days (**Figure 2B**), both with a purity of 90% or more. After being intervened by SCU, C_18_H_17_NO_6_ or the combination for 48 h, the cell viability was detected by MTT assay.

Firstly, as SCU dose increased, the cell viability of astrocytes declined gradually, and to the lowest level (about 50% of the normal state) when the dose reached 300~400 μM (**Figure 2C**). Moreover, the further increase in SCU dose did not continue to reduce the cell viability of astrocytes (**Figure 2C**). Additionally, the 50% inhibition rate (IC_50_) of SCU in astrocytes was 1,255 μM with 95% CI 562.7 to 2,799 μM (**Figure 2D**), which was higher than that in glioma cells (250.4~294.9 μM), indicating that although SCU was ever toxic to astrocytes, the toxicity of its effective dose to successfully suppress glioma cell proliferation was relatively low on astrocytes.

As previously reported (32), C_18_H_17_NO_6_ has low toxicity to astrocytes with IC_50_ 14.55 μM, which was much higher than that in glioma cells (4 to 7 μM). What was the toxicity of the combination? Next, we explored the toxicity of SCU and its combination with C_18_H_17_NO_6_ in astrocytes. Our results showed that there was no significant difference in cell viability of astrocytes between the SCU 100 or 200 μM groups and control group (0.163% DMSO), suggesting SCU 100 or 200 μM was nontoxic (**Figure 2E**). However, compared with the control group, SCU 400 μM significantly reduced cell viability of astrocytes (**Figure 2E**, *p* = 0.021). Additionally, SCU could induce the apoptosis of astrocytes, but the maximum apoptosis rate at SCU 400 μM was not more than 10.5% (**Figures 2G, H** **)**, which was far less than that in glioma cells (about 30%, see below). In this study, the embedded dose of C_18_H_17_NO_6_ was 2 to 3 μM, which was only 13.7%~20% of IC_50_ in astrocytes (32). Consistent with the previous report (32), C_18_H_17_NO_6_ 3 μM harbored no toxic effect on astrocytes (**Figure 2E**). When both drugs were combined, only SCU 400 μM plus C_18_H_17_NO_6_ 3 μM significantly decreased cell viability of astrocytes compared with the control group (**Figure 2E**, *p* = 0.031). Furthermore, compared with the SCU alone groups, cell viability of astrocytes showed an increasing trend in the combination groups, but the difference was not statistically significant (**Figure 2E**, *p* > 0.05). Also, the apoptosis rate induced by the combination groups was very low, not more than 10.5% (**Figures 2G, H** **)**. The above results pointed out that the toxicity of combination to astrocytes originated from high dose of SCU, and C_18_H_17_NO_6_ 3 μM was nontoxic and also did not exacerbate the toxicity of SCU.

In the course of chemotherapy for brain tumors, we should consider the toxicity of drugs not only to glial cells but also neurons. Thus, we also examined the toxicity of SCU and its combination with C_18_H_17_NO_6_ 3 μM to cortical neurons. The results showed that with the increase of SCU, cell viability of cortical neurons declined (**Figure 2F**). Moreover, the difference was significant between the SCU 200 or 400 μM groups and control group (**Figure 2F**, *p* < 0.05). In addition, C_18_H_17_NO_6_ 3 μM also reduced cell viability of cortical neurons, but there was no significant difference between the C_18_H_17_NO_6_ 3 μM group and control group (**Figure 2F**, *p* > 0.05). Furthermore, although the SCU 200 μM plus C_18_H_17_NO_6_ 3 μM and SCU 400 μM plus C_18_H_17_NO_6_ 3 μM significantly decreased neuronal viability compared with the control group (**Figure 2F**, *p* < 0.05), there was no significant difference in neuronal viability between the SCU alone groups and the combination groups (**Figure 2F**, *p* > 0.05). These results also illustrated that C_18_H_17_NO_6_ 3 μM was nontoxic and did not amplify the toxicity of SCU to cortical neurons. Among the combination of SCU and C_18_H_17_NO_6_ 3 μM, the toxicity to neurons was from the high dose of SCU.

In summary, SCU and C_18_H_17_NO_6_ harbored significant inhibitory effect on glioma cells, but both and their combination were less toxic to normal cells. The notion suggested that the combination of multiple chemotherapeutic drugs can reduce not only the toxicity of single drug used in large doses but also the drug resistance (32). Consequently, the next study focused on the role of the combination of SCU and C_18_H_17_NO_6_ in glioma cells.

### Effect of Scutellarin and Its Combination With C_18_H_17_NO_6_ on the Viability of Glioma Cells

**Figure 3:**
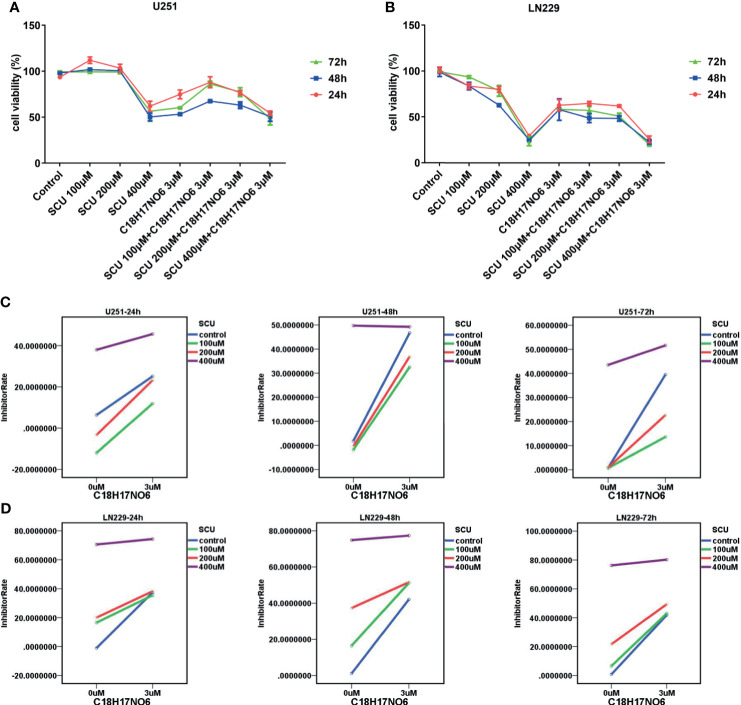
Effect of scutellarin and its combination with C_18_H_17_NO_6_ on the viability of glioma cells. **(A, B)** Effect of SCU and its combination with C_18_H_17_NO_6_ 3 μM on the viability of U251 and LN229 cells over time. **(C, D)** The interaction diagrams of SCU and C_18_H_17_NO_6_ in U251 and LN229 cells. The *p*-values of the interaction in these diagrams (from left to right, from top to bottom) were 0.005, 0.000, 0.000, 0.000, 0.000, and 0.000, respectively. Control group: Con, 0.163% DMSO. Cell viability = (As − Ab)/(Ac − Ab) * 100%. As, absorption of drug-added wells (experimental groups); Ac, absorption of solvent-added wells (control group); Ab, absorption of blank control wells, which are the wells containing medium and CCK-8 reagent.

The cell viability of U251 and LN229 cells intervened by SCU and its combination with C_18_H_17_NO_6_ 3 μM for 24, 48, and 72 h was detected by cell counting kit-8 (CCK-8; DOJINDO, Kumamoto, Japan). The results demonstrated that SCU possessed a concentration-dependent effect on glioma cells (**Figures 3A–D**). For U251 cells, it restrained the cell viability only at high concentration (400 μM), but at low concentration (100 μM), it harbored a certain effect on the proliferation. However, the proliferation effect of SCU at low concentration could be reversed by C_18_H_17_NO_6_ 3 μM (**Figure 3A**). For LN229 cells, the inhibition rate was raised with the increase of SCU concentration, and the inhibitory effect of it on LN229 was enhanced by C_18_H_17_NO_6_ 3 μM (**Figure 3B**). Simultaneously, there was an interaction between them (**Figures 3C, D** **)**. Furthermore, the inhibitory effect of SCU and its combination with C_18_H_17_NO_6_ 3 μM on U251 and LN229 was time dependent, but the inhibition rate reached the maximum at 48 h (**Figures 3A, B** **)**.

### Effect of Scutellarin and Its Combination With C_18_H_17_NO_6_ on the Clone Formation of Glioma Cells

**Figure 4:**
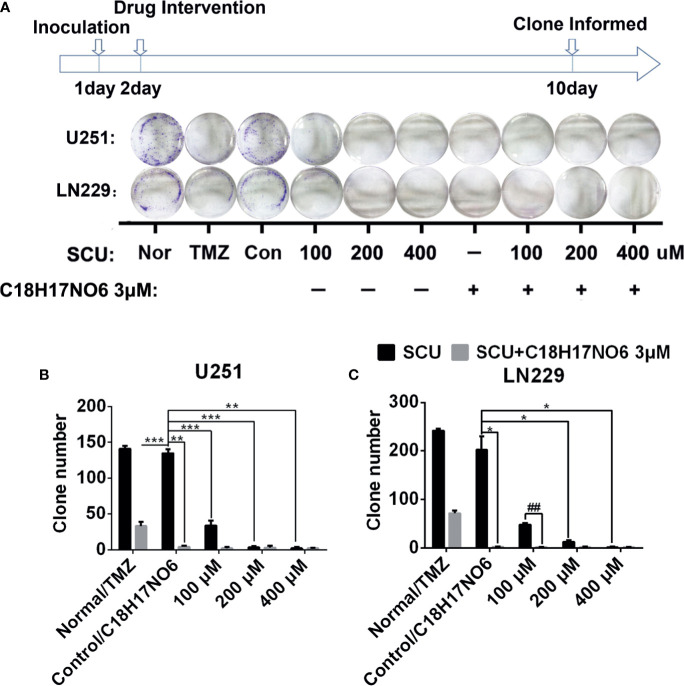
Effect of scutellarin and its combination with C_18_H_17_NO_6_ on the clone formation of glioma cells. **(A)** The pictures show the effect of SCU and its combination with C_18_H_17_NO_6_ 3 μM on the clone formation of U251 and LN229 cells. **(B**, **C)** The quantity of formed clones. The clone with more than 50 cells was counted as one clone. TMZ, the positive control drug—temozolomide 200 μM group. Nor, normal group (cells) that did not receive any intervention. “*” *vs.* control (Con, 0.163% DMSO), “^#^” SCU *x* vs. SCU *x* + C_18_H_17_NO_6_ 3 μM, *p < 0.05, **/^##^ *p* < 0.01, ***p < 0.001 (*n* = 3).

The cloning ability of U251 and LN229 cells was tested by plate clone formation assay. Cells were inoculated on the first day. After cell adherence overnight, the cells were intervened with SCU and its combination with C_18_H_17_NO_6_ 3 μM and the drug intervention lasted for 9 days. On the 10th day, 0.5% crystal violet (Beyotime, Jiangsu, China) staining was performed, and the number of clones was counted. Meanwhile, the medium was changed every 3 days. We found that compared with control group (0.163% DMSO), the number of clones of U251 and LN229 cells in SCU groups decreased significantly (*p* < 0.05) (**Figure 4**). Moreover, the higher the SCU dose, the fewer the clone number (**Figure 4**). Additionally, compared with the control group, the cloning of U251 and LN229 cells was also inhibited by C_18_H_17_NO_6_ 3 μM, and the difference was statistically significant (*p* < 0.05) (**Figure 4**). Since the inhibition of SCU was time dependent, its effect on clone formation reached a peak at a concentration of 200 μM, so the addition of C_18_H_17_NO_6_ 3 μM showed no effect (**Figure 4**). It was only observed that the combination of SCU 100 μM and C_18_H_17_NO_6_ 3 μM could further restrained the cloning formation of U251 and LN229 cells in comparison with SCU 100 μM group (*p* < 0.05) (**Figure 4**). Besides, compared with the positive control drug-temozolomide (Sigma, St. Louis, MO, USA), SCU and its combination with C_18_H_17_NO_6_ 3 μM further suppressed the clone formation of U251 and LN229 cells (**Figure 4**), demonstrating that SCU and its combination with C_18_H_17_NO_6_ suppressed the proliferation of U251 and LN229 cells more effectively than temozolomide.

### Effect of Scutellarin and Its Combination With C_18_H_17_NO_6_ on Proliferation of Glioma Cells

**Figure 5:**
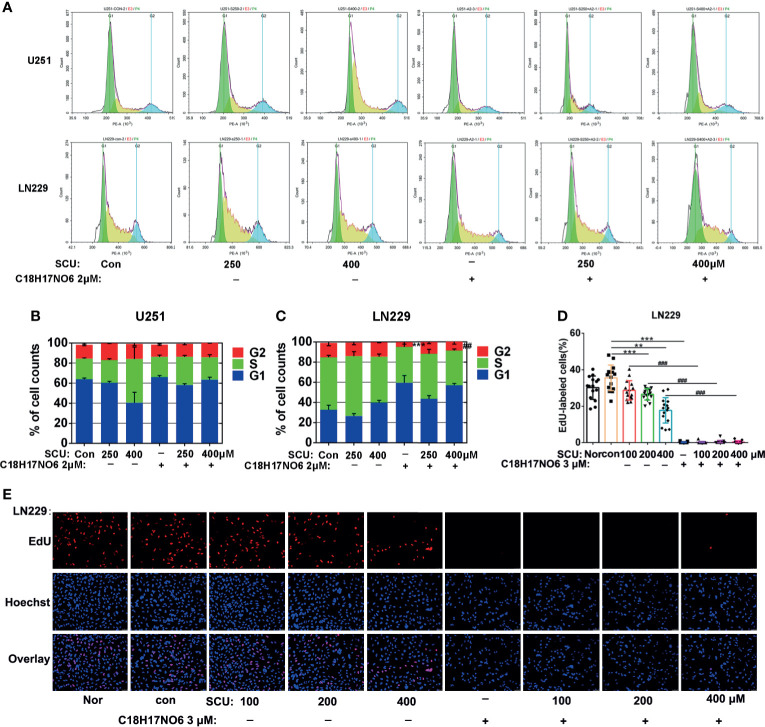
Effect of scutellarin and its combination with C_18_H_17_NO_6_ on proliferation of glioma cells. **(A)** The diagrams show the cell cycles of U251 and LN229 cells after intervention by SCU and its combination with C_18_H_17_NO_6_ 2 μM; **(B**, **C)** Quantification of the ratio of U251 and LN229 cells in G1, S, and G2 phases (*n* = 3). **(D)** Fluorescence images show the effect of SCU and its combination with C_18_H_17_NO_6_ 3 μM on the proliferation of LN229 cells after intervention for 48 h; **(E)** The proliferation rate of LN229 cells after intervention by SCU and its combination with C_18_H_17_NO_6_ 3 μM for 48 h (*n* = 15). The proliferation rate was equal to the number of EdU-positive cells (red)/Hoechst-positive cells (blue) × 100%. Nor, normal group (cells) that did not receive any intervention. “*” *vs.* control (Con, 0.163% DMSO), “^#^” SCU *x vs.* SCU *x* + C_18_H_17_NO_6_ 2/3 μM, **/^##^ *p* < 0.01, ***/^###^ *p* < 0.001.

Here, we detected the proliferation of glioma cells by EdU incorporation assay and cell cycle. Firstly, the cell cycle of glioma cells after 48 h of intervention by SCU and its combination with C_18_H_17_NO_6_ 2 μM was detected by flow cytometry (ACEA). The results demonstrated that with the rise of SCU concentration, the proportion of LN229 cells in G1 phase increased after intervention for 48 h, while U251 cells in G1 phase did not differ between groups (**Figures 5A–C**). For the proportion of U251 and LN229 cells in G2 phase, there was no significant difference between the control group (0.153% DMSO) and the SCU groups (*p* > 0.05) (**Figures 5A–C**). In addition, compared with the control group, C_18_H_17_NO_6_ 2 μM raised the proportion of U251 and LN229 cells in G1 phase but reduced the ratio of U251 and LN229 cells in G2 and S phases, in which the ratio of LN229 cells in G2 phase was declined significantly (*p* < 0.05) (**Figures 5A–C**). Compared with the SCU alone groups, the combination of SCU and C_18_H_17_NO_6_ 2 μM reduced the ratio of U251 and LN229 cells in G2 phase. What is more, for the decrease in LN229 cells in G2 phase, the difference between SCU 400 μM and SCU 400 μM plus C_18_H_17_NO_6_ 2 μM group was statistically significant (*p* < 0.05) (**Figures 5A–C**). In the EdU incorporation assay, we found that after intervention for 48 h, SCU controlled the proliferation of LN229 cells in a concentration-dependent manner (**Figures 5D, E** **)**. In addition, C_18_H_17_NO_6_ 3 μM significantly inhibited the proliferation of LN229 cells and improved this inhibitory effect of SCU (**Figures 5D, E** **)**.

### Effect of Scutellarin and Its Combination With C_18_H_17_NO_6_ on Apoptosis of Glioma Cells

The apoptosis of U251 and LN229 cells, induced by SCU and its combination with C_18_H_17_NO_6_ for 48 h, was detected by both terminal-deoxynucleoitidyl transferase-mediated nick end labeling (TUNEL) staining and flow cytometry.

**Figure 6:**
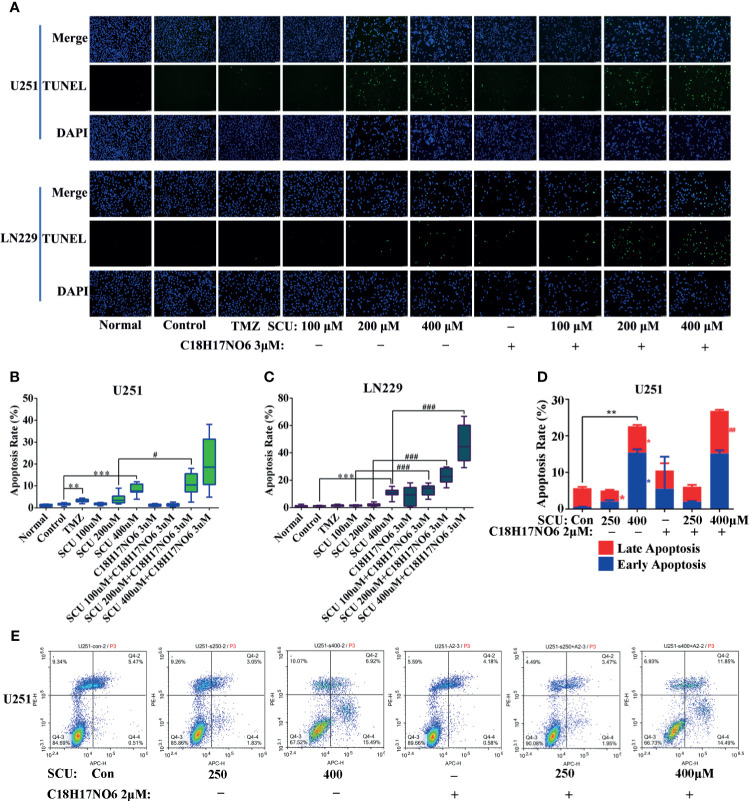
Effect of scutellarin and its combination with C_18_H_17_NO_6_ on apoptosis of glioma cells. **(A)** Fluorescence images showed the apoptosis of U251 and LN229 cells induced by SCU and its combination with C_18_H_17_NO_6_ 3 μM. **(B**, **C)** Quantification of apoptosis rate of U251 and LN229 cells induced by SCU and its combination with C_18_H_17_NO_6_ 3 μM (*n* =10). Apoptosis rate = TUNEL-positive cells (green)/Hoechst-positive cells (blue) × 100%. TMZ, the positive control group (temozolomide 200 μM). Normal, normal group (cells) that did not receive any intervention. **(D**, **E)** The apoptosis of U251 cells induced by SCU and its combination with C_18_H_17_NO_6_ 2 μM by flow cytometry (*n* = 3). “*” *vs.* control (Con, 0.153% DMSO), “^#^” SCU *x vs.* SCU *x* + C_18_H_17_NO_6_ 2/3 μM, */^#^ *p* < 0.05, **/^##^ *p* < 0.01, ***/^###^ *P* < 0.001.

TUNEL staining demonstrated that compared with control group (0.163% DMSO), the apoptosis rate of U251 and LN229 was elevated with the increase of SCU concentration, but only the difference between the SCU 400 μM group and control group had statistical significance (*p* < 0.001) (**Figures 6A–C**). Moreover, compared with the control group, the apoptosis rate of U251 and LN229 were also increased by C_18_H_17_NO_6_ 3 μM, but the difference was not statistically significant (*p* > 0.05) (**Figures 6A–C**). When SCU was combined with C_18_H_17_NO_6_ 3 μM, the apoptosis rate of U251 and LN229 cells was further markedly elevated in comparison with the SCU groups at the same SCU concentration alone (*p* < 0.05), indicating that C_18_H_17_NO_6_ 3 μM boosted the apoptosis of U251 and LN229 cells induced by SCU (**Figures 6A–C**). In addition, compared with the positive control group (temozolomide 200 μM), SCU 200 μM or 400 μM plus C_18_H_17_NO_6_ 3 μM further induced U251 cell apoptosis (**Figures 6A, B** **)**, and SCU 400 μM and SCU plus C_18_H_17_NO_6_ 3 μM led to LN229 cell apoptosis more effectively (**Figures 6A, C** **)**, indicating that the combined effect of SCU and C_18_H_17_NO_6_ on apoptosis of U251 and LN229 cells was better than that of temozolomide.

In addition, flow cytometry detection held the similar results with TUNEL staining, which elaborated that with the increase in the SCU dosage, the early apoptosis rate and late apoptosis rate of U251 went up after 48 h of intervention, and the difference was statistically significant in comparison with the control group (0.153% DMSO) (*p* < 0.05) (**Figures 6D, E** **)**. Besides, compared with the control group, C_18_H_17_NO_6_ 2 μM also promoted apoptosis of U251 cells, but the difference was not statistically significant (*p* > 0.05) (**Figures 6D, E** **)**. Moreover, SCU plus C_18_H_17_NO_6_ 2 μM further induced the late apoptotic of U251 in comparison with SCU alone groups, and the difference between the SCU 400 μM plus C_18_H_17_NO_6_ 2 μM group and SCU 400 μM group was statistically significant (*p* < 0.05) (**Figures 6D, E** **)**.

### Effect of Scutellarin and Its Combination With C_18_H_17_NO_6_ on Lateral Migration of Glioma Cells

**Figure 7:**
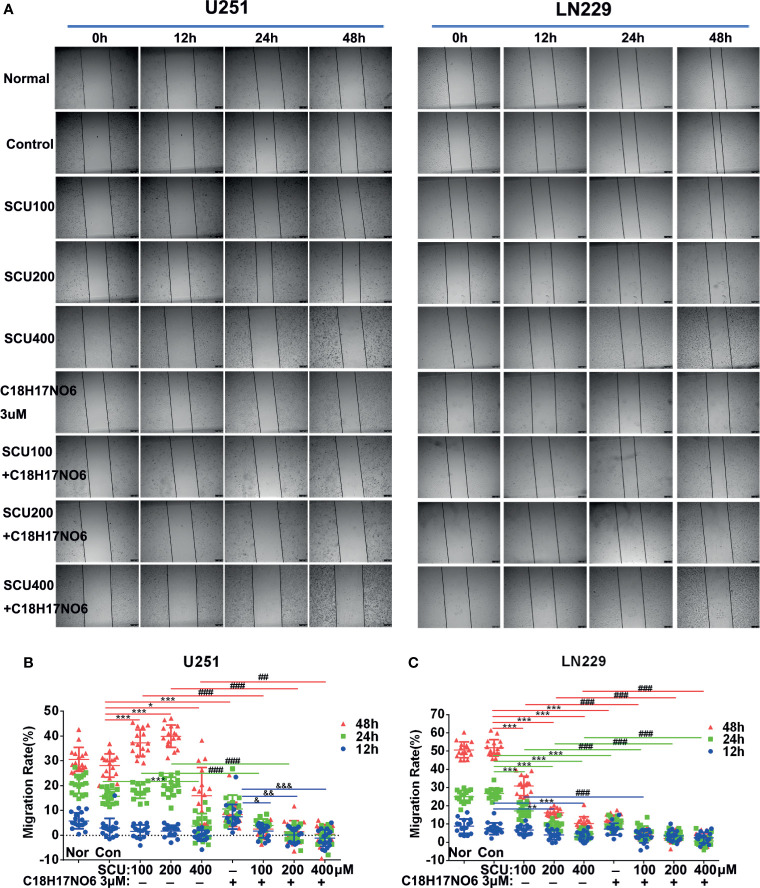
Effect of scutellarin and its combination with C_18_H_17_NO_6_ on lateral migration of glioma cells. **(A)** Images show the effect of SCU and its combination with C_18_H_17_NO_6_ 3 μM on the lateral migration of U251 and LN229 cells. **(B**, **C)** Quantification of the lateral migration rate. Blue, green and red represent the migration rate at 12, 24, and 48 h, respectively. Migration rate = (the shortest distance at 0 h − *X*h)/the shortest distance at 0 h * 100%. Nor, normal group (cells) that did not receive any intervention. “*” *vs.* control (Con, 0.163% DMSO), “^#^” SCU *x vs.* SCU *x* + C_18_H_17_NO_6_ 3 μM, “&” C18H17NO6 3 μM vs. SCU x + C18H17NO6 3 μM, */^&^ *p* < 0.05, **/^##^/^&&^ *p* < 0.01, ***/^###^/^&&&^ *p* < 0.001 (*n* = 16).

The lateral migration ability of U251 and LN229 cells administrated with SCU and its combination with C_18_H_17_NO_6_ was measured by wound-healing assay. The results revealed that with the increase of SCU, the lateral migration rate of U251 and LN229 cells was significantly lower than that of the control group (0.163% DMSO) after intervention for 24 and 48 h (*p* < 0.05) (**Figure 7**); however, at 12 h of administration, the lateral migration of U251 cells was not significantly different from that of the control group, but it was significantly inhibited in LN229 cells (*p* < 0.05) (**Figure 7**). Besides, under the same intervention conditions, the migration rate of U251 and LN229 cells increased over time (**Figure 7**). At the same time, C_18_H_17_NO_6_ 3 μM also restrained the migration of U251 and LN229 cells, and the difference at 24 and 48 h of intervention was statistically significant in comparison with the control group (*p* < 0.05) (**Figure 7**). Furthermore, after intervention for 24 and 48 h, the lateral migration rate of U251 and LN229 cells in SCU plus C_18_H_17_NO_6_ 3 μM groups was significantly lower than that in the corresponding SCU alone groups (*p* < 0.05), which illustrated that the inhibitory effect of SCU on lateral migration of U251 and LN229 cells was promoted by C_18_H_17_NO_6_ (**Figure 7**).

### Effect of Scutellarin and Its Combination With C_18_H_17_NO_6_ on Vertical Migration of Glioma Cells

**Figure 8:**
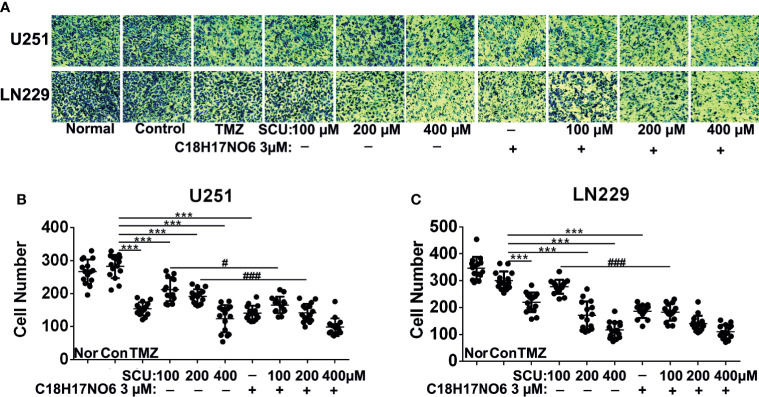
Effect of scutellarin and its combination with C_18_H_17_NO_6_ on vertical migration of glioma cells. **(A)** Images show the U251 and LN229 cells that vertically migrated to the bottom of the chamber after intervention by SCU and its combination with C_18_H_17_NO_6_ 3 μM for 48 h. **(B**, **C)** Quantification of the U251 and LN229 cells that vertically migrated to the bottom of the chamber. TMZ, the positive control group (temozolomide 200 μM); Nor, normal group (cells) that did not receive any intervention. “*” *vs.* control (Con, 0.163% DMSO), “^#^” SCU *x vs.* SCU *x* + C_18_H_17_NO_6_ 3 μM, ^#^ *p* < 0.05, ***/^###^ *p* < 0.001 (*n* = 15).

The vertical migration ability of U251 and LN229 cells intervened by SCU and its combination with C_18_H_17_NO_6_ for 48 h was tested by Transwell assay. As the concentration of SCU increased, the number of U251 and LN229 cells that vertically migrated to the bottom of the chamber was significantly lower than that of the control group (0.163% DMSO; *p* < 0.05) (**Figure 8**). Similarly, compared with the control group, C_18_H_17_NO_6_ 3 μM also substantially suppressed the vertical migration of both cells (*p* < 0.05) (**Figure 8**). Moreover, the number of the migrated U251 and LN229 cells in the SCU plus C_18_H_17_NO_6_ 3 μM groups was smaller than that in the corresponding SCU groups. As shown in **Figure 8**, the number of migrated U251 cells significantly decreased in the SCU 200 μM plus C_18_H_17_NO_6_ 3 μM group when compared with the SCU 200 μM group (*p* < 0.05), while in LN229 cell, the difference between the SCU 100 μM plus C_18_H_17_NO_6_ 3 μM group and SCU 10 0μM group was statistically significant (*p* < 0.05). The above indicated that C_18_H_17_NO_6_ aggravated the inhibition of SCU on vertical migration of U251 and LN229 cells. Furthermore, compared with the positive control group, SCU 400 μM and its combination with C_18_H_17_NO_6_ 3 μM further restrained the vertical migration of U251 cells (**Figures 8A, B** **)**, and SCU 200 μM and 400 μM, C_18_H_17_NO_6_ 3 μM as well as their combination all inhibited the vertical migration of LN229 cells more efficiently (**Figures 8A, C** **)**, suggesting that the inhibitory effect of SCU and its combination with C_18_H_17_NO_6_ on migration of U251 and LN229 cells was better than that of temozolomide.

### Presenilin 1 Was Downregulated in Glioma, While It Was Upregulated by Scutellarin and Its Combination With C_18_H_17_NO_6_ *In Vitro*

**Figure 9:**
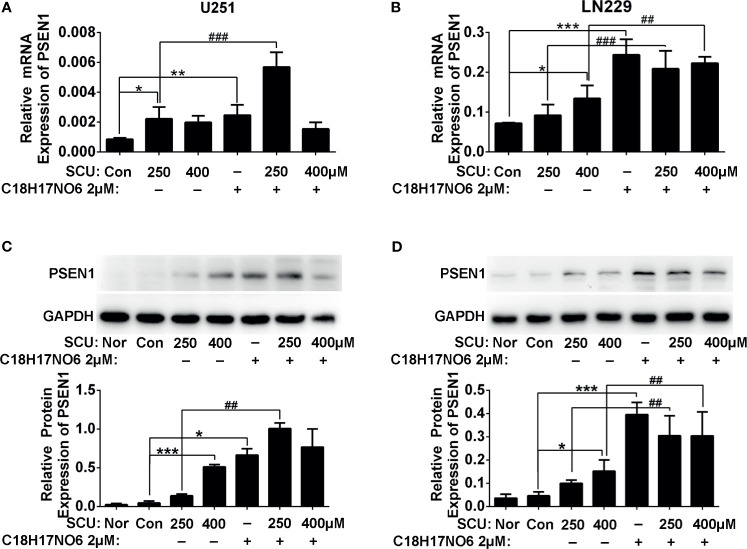
The PSEN1 expression in U251 and LN229 cells after intervention by scutellarin and its combination of C_18_H_17_NO_6_ for 48 h. **(A)** The mRNA expression of PSEN1 in U251 cell. **(B)** The mRNA expression of PSEN1 in LN229 cell. **(C)** The protein expression of PSEN1 in U251 cell. **(D)** The protein expression of PSEN1 in LN229 cell. “*” *vs.* control (0.153% DMSO), “^#^” SCU *x vs.* SCU *x* + C_18_H_17_NO_6_ 2 μM, *p < 0.05, **/^##^ *p* < 0.01, ***/^###^ *p* < 0.001 (*n* = 3).

On the one hand, compared with normal tissue, PSEN1 expression in glioma was downregulated. As is shown in **Supplementary Figure S1**, in 166 glioma samples, PSEN1 was changed in 18% of the samples, in which the expression was mainly downregulated. Moreover, immunoblotting assay also demonstrated that the PSEN1 protein level in glioma cells was less than that in normal astrocytes and neurons (**Supplementary Figure S5**). On the other hand, the mRNA and protein expression of PSEN1 in U251 and LN229 cells was upregulated by SCU and its combination with C_18_H_17_NO_6_. For mRNA level, compared with the control group (0.153% DMSO), the PSEN1 mRNA in U251 cell was significantly increased in the SCU 250 μM group and C_18_H_17_NO_6_ 2 μM group (**Figure 9A** and **Supplementary Figure S2**, *p* < 0.05). Besides, the SCU 250 μM plus C_18_H_17_NO_6_ 2 μM could further raise the PSEN1 mRNA level in U251 cell in comparison with the SCU 250 μM (**Figure 9A** and **Supplementary Figure S2**, *p* < 0.001). In LN229 cell, compared with the control group, SCU significantly increased the expression of PSEN1 mRNA, and the difference between the SCU 400 μM and control group was statistically significant (**Figure 9B** and **Supplementary Figure S3**, *p* < 0.05). Moreover, the expression of PSEN1 mRNA in LN229 cell also was significantly upregulated by C_18_H_17_NO_6_ 2 μM in comparison with the control group (**Figure 9B** and **Supplementary Figure S3**, *p* < 0.001). Compared with SCU alone, SCU (whether 250 or 400 μM) plus C_18_H_17_NO_6_ 2 μM further elevated PSEN1 mRNA level in LN229 cell (**Figure 9B** and **Supplementary Figure S3**, *p* < 0.01). In addition, the PSEN1 protein level was similar to its mRNA level (**Figures 9C, D** **)**. All the above indicated that SCU and its combination with C_18_H_17_NO_6_ might upregulate the PSEN1 expression to suppress the proliferation and the migration and induce the apoptosis of glioma cells.

### The Activity of the PI3K-AKT Signaling Pathway Was Repressed by Scutellarin and Its Combination With C_18_H_17_NO_6_ *In Vitro*

**Figure 10:**
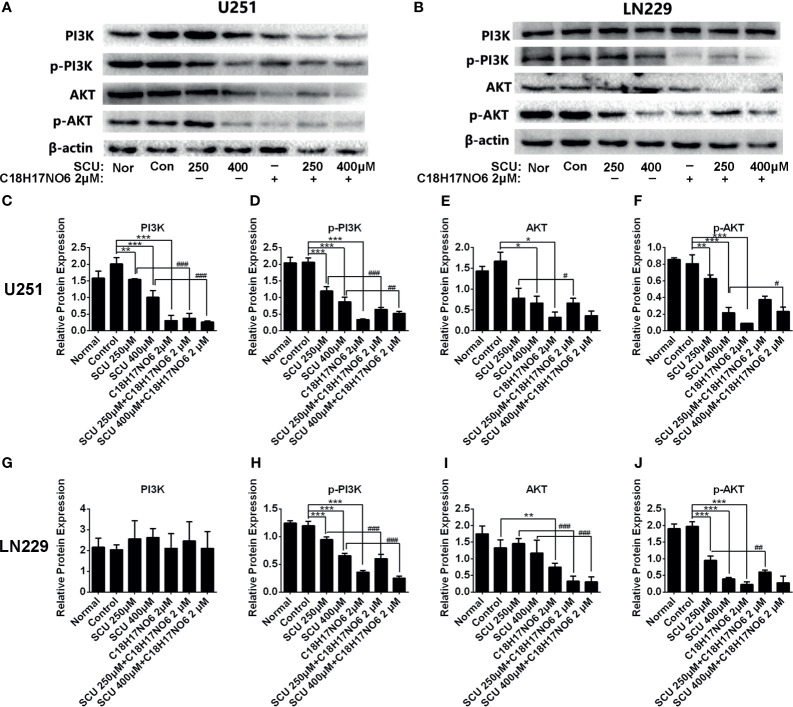
The protein expression of PI3K, p-PI3K, AKT, and p-AKT in U251 and LN229 cells after intervention by scutellarin and its combination of C_18_H_17_NO_6_. **(A, B)** Protein bands showed the change in the protein expression of PI3K, p-PI3K, AKT, and p-AKT in U251 and LN229 cells after intervention by scutellarin and its combination of C_18_H_17_NO_6_ for 48h; **(C–J)** Quantification of the protein expression of PI3K, p-PI3K, AKT, and p-AKT in U251 and LN229 cells after intervention for 48 h Nor, normal group (cells) that did not receive any intervention. “*” vs. control (Con, 0.153% DMSO), “^#^” SCU *x vs.* SCU *x* + C_18_H_17_NO_6_ 2 μM, “*/^#^ *p* < 0.05, **/^##^ *p* < 0.01, ***/^###^ *p* < 0.001 (*n* = 3).

In U251 cell, compared with the control group (0.153% DMSO), SCU significantly downregulated the protein expression of PI3K, p-PI3K, AKT, and p-AKT (*p* < 0.05) (**Figures 10A, C–F**). Moreover, the higher the dose of SCU, the lower the expression level of these four proteins (**Figures 10A, C–F**). Additionally, the expression of PI3K, p-PI3K, AKT, and p-AKT in U251 cell was also significantly reduced by C_18_H_17_NO_6_ 2 μM in comparison with that in the control group (*p* < 0.05) (**Figures 10A, C–F**). Furthermore, the PI3K, p-PI3K, AKT, and p-AKT protein level was less in the SCU plus C_18_H_17_NO_6_ 2 μM groups than that in the corresponding SCU alone groups (**Figures 10A, C–F**).

In LN229 cell, the protein expression of PI3K was not affected by SCU and its combination with C_18_H_17_NO_6_ (**Figures 10B, G** **)**. However, compared with the control group, SCU could significantly decrease the expression of p-PI3K and p-AKT proteins in LN229 cell (*p* < 0.05), and the expression of these two proteins dropped down by degrees with the increase of SCU concentration (**Figures 10B, H, J** **)**, which, but, did not affect the expression of AKT protein (**Figures 10B, I** **)**. The level of p-PI3K, AKT, and p-AKT protein in LN229 cells was also significantly downregulated by C_18_H_17_NO_6_ 2 μM in comparison with the control group (*p* < 0.05) (**Figures 10B, H–J**). Simultaneously, SCU plus C_18_H_17_NO_6_ 2 μM further reduced the expression of p-PI3K, AKT, and p-AKT protein in comparison with the corresponding SCU alone (**Figures 10B, H–J**).

### Scutellarin and Its Combination With C_18_H_17_NO_6_ Suppressed the Growth of Glioma *In Vivo*, Also Relating to Inactivating PI3K-AKT Signaling

**Figure 11:**
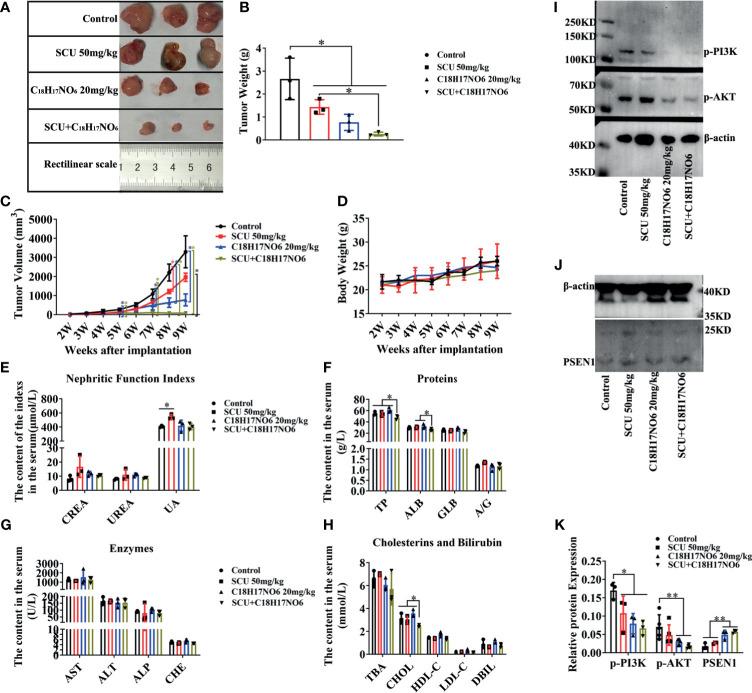
Scutellarin and its combination with C_18_H_17_NO_6_ suppressed the growth of glioma *in vivo*, also relating to inactivating PI3K-AKT signaling. **(A, B)** The tumor size and weight after 5 weeks administration with SCU 50 mg/kg, C_18_H_17_NO_6_ 20 mg/kg, and their combination. **(C, D)** The tumor volume and the body weight of nude mice after implantation of U251 cells for 2 to 9 weeks. **(E–H)** Quantification of indicators that reflecting the liver and kidney function, including the creatinine (CREA) and urea nitrogen (UREA), uric acid (UA), total protein (TP), albumin (ALB), globulin (GLB), albumin/globulin ratio (A/G), alanine aminotransferase (ALT), aspartate aminotransferase (AST), alkaline phosphatase (ALP), cholinesterase (CHE), total bile acid (TBA), cholesterol (CHOL), low-density lipoprotein cholesterol (LDL-C), high-density lipoprotein cholesterol (HDL-C), and direct bilirubin (DBIL). **(I–K)** The protein level of p-PI3K, p-AKT, and PSEN1 in the tumor tissue after 5 weeks drug administration. *p < 0.05, **p < 0.01 (*n* = 3–5).

To further validate the *in vitro* observations, we established the *in vivo* xenograft tumor model of glioma and injected scutellarin 50 mg/kg, C_18_H_17_NO_6_ 20 mg/kg, and the combination (scutellarin 50 mg/kg and C_18_H_17_NO_6_ 20 mg/kg) intraperitoneally into nude mice to observe the growth of tumor. Consistent with the *in vitro* results, scutellarin 50 mg/kg and C_18_H_17_NO_6_ 20 mg/kg inhibited tumor growth *in vivo*, and the tumor size, weight, and volume derived from scutellarin 50 mg/kg and C_18_H_17_NO_6_ 20 mg/kg groups were dramatically smaller than those of the control group (10% DMSO) (**Figures 11A–C**). Moreover, the combination held a better treatment effect (**Figures 11A–C**). However, there is no difference in the body weight of nude mice among these four groups (**Figure 11D**), suggesting that scutellarin 50 mg/kg, C_18_H_17_NO_6_ 20 mg/kg, or the combination harbored no effect on the growth of mice.

In addition, the toxicity of these drugs was also evaluated. Here, the indicators reflecting the liver and kidney function were detected. Our results demonstrated that there was no difference in the creatinine (CREA) and urea nitrogen (UREA) among these four groups, but scutellarin 50 mg/kg therapy could slightly increase the uric acid (UA) in the serum of nude mice (**Figure 11E**). Furthermore, the total protein (TP), albumin (ALB), and cholesterol (CHOL) in the combination of scutellarin 50 mg/kg and C_18_H_17_NO_6_ 20 mg/kg group were reduced in comparison with the control, SCU 50 mg/kg, and C_18_H_17_NO_6_ 20 mg/kg groups (**Figures 11F, H** **)**. Nevertheless, globulin (GLB), albumin/globulin ratio (A/G), alanine aminotransferase (ALT), aspartate aminotransferase (AST), alkaline phosphatase (ALP), cholinesterase (CHE), total bile acid (TBA), low-density lipoprotein cholesterol (LDL-C), high-density lipoprotein cholesterol (HDL-C), and direct bilirubin (DBIL) in the serum, which all reflected the liver function, did not differ among the four groups (**Figures 11F–H**). These results indicated that SCU 50 mg/kg might impair the kidney function and the combination of SCU 50 mg/kg and C_18_H_17_NO_6_ 20 mg/kg might slightly damage the liver function, but the toxicity of SCU 50 mg/kg or C_18_H_17_NO_6_ 20 mg/kg alone on liver was not observed.

Finally, we quantified the protein level of PSEN1/PI3K-AKT signaling axis in the tumor tissue. Just like the *in vitro* results, the expression of PSEN1 protein was upregulated by scutellarin 50 mg/kg, C_18_H_17_NO_6_ 20 mg/kg, and their combination (**Figures 11J, K** **)**. As is known to all, the phosphorylated form of protein is its functional form, so we only detected the p-PI3K and p-AKT. As expected, the protein level of p-PI3K and p-AKT was downregulated by scutellarin 50 mg/kg, C_18_H_17_NO_6_ 20 mg/kg, and their combination (**Figures 11I, K** **)**. Therefore, the antiglioma effect of scutellarin, C_18_H_17_NO_6_, and their combination might be associated with upregulated PSEN1 and the deactivation of PI3K-AKT signaling.

## Discussion

In this study, we first found that single-agent SCU and C_18_H_17_NO_6_ suppressed glioma cell proliferation and migration in a concentration-dependent manner. Under intervention by the combination, the proliferation and migration ability of U251 and LN229 cells were further reduced, and the apoptosis was also induced, that is, the two drugs exert a synergistic effect. Moreover, SCU and its combination with C_18_H_17_NO_6_ also suppressed the growth of glioma *in vivo*. More importantly, the effect of SCU and its combination with C_18_H_17_NO_6_ on glioma cells was better than positive control-temozolomide, but the toxicity was low. Finally, the mechanism might be associated with the upregulated PSEN1 repressing the activation of PI3K-AKT signaling.

Scutellarin, as an antitumor drug, has been reported in a large number of preclinical studies. Scutellarin and its derivatives possessed antiproliferation and apoptotic effects on a variety of cancer cell lines, consisting of MCF-7, HCT116, PC-3, and HepG2 cells but exerted very low toxicity to normal hepatocyte L-O_2_ cells (33). In terms of mechanism, SCU could directly interact with PKM2 to inhibit its cytoplasmic activity and reduce glycolysis; moreover, SCU might also regulate the proteins related to cell cycle and apoptosis by activating MEK/ERK/PIN1 signaling, thereby promoting PKM2 nuclear translocation (34). SCU suppressed not only tumor cell proliferation but also migration and invasion. Zhu and his colleagues found that scutellarin inhibited the proliferation, angiogenesis, and metastasis of colorectal cancer cells by targeting ephrinb2 signal (35). Similarly, as a newly developed antitumor drug, C_18_H_17_NO_6_ has been reported to harbor a strong antitumor effect *in vitro* and *in vivo* (Patent No.: 201710388136.8) (32). Consistent with previous studies, we also found that single-agent SCU and C_18_H_17_NO_6_, with low toxicity (32), inhibited the proliferation/growth and migration of glioma in the present study. In addition, many researchers found that scutellarin could facilitate the communication between activated microglia and reactive astrocytes in the cerebral cortex, suggesting that scutellarin could cross the blood-brain barrier (BBB) (36–39). Although whether C_18_H_17_NO_6_ can pass through the BBB remained to be specifically illustrated, it could exactly penetrate blood through the vessels around tumor cells to inhibit tumor proliferation *in vivo*, as our *in vivo* data of subcutaneous xenograft showed. Therefore, scutellarin and C_18_H_17_NO_6_ might be the candidate antiglioma drugs in the future.

It is generally acknowledged that the combination of multidrug with low dose cannot only reduce the drug resistance of cancer but also abate the toxicity caused by the large-dose use of single drug (40). Therefore, we studied the combined effect of the two drugs on glioma and found that the two drugs synergistically inhibited the cell viability, clone formation and proliferation, induced the apoptosis, and suppressed the migration laterally and vertically of U251 and LN229 cells *in vitro*. Moreover, they held a similar effect *in vivo*. Studies have reported that SCU not only possessed antitumor effect but also could improve the sensitivity of cancer cells to chemotherapeutic drugs and reduce their toxicity. SCU combined with bleomycin could further inhibit the cell viability of H22 and MRC-5 cells and promote their apoptosis (41). What is more, SCU could extend the survival time of mice bearing H22 cell tumor and reduce bleomycin-induced pulmonary fibrosis *in vivo* (41). In addition, SCU could be used as a sensitizer for cisplatin. In ovarian cancer (42), nonsmall-cell lung cancer (NSCLC) (43), and prostate cancer (44), SCU interacted with cisplatin to promote cell apoptosis, sensitize the response of cancer cells to cisplatin, and debase its toxicity. At the same time, C_18_H_17_NO_6_ could also serve as an anticancer drug, a composition of combined anticancer drug, a chemopreventive drug for cancer, an anticancer health product, an antimutagen, etc. (Patent No. 201710388136.8). Therefore, the combination of SCU and C_18_H_17_NO_6_ can exert more effective antiglioma effect. In addition, we found that the effect of SCU and its combination with C_18_H_17_NO_6_ on glioma cells was better than the clinical standard drug-temozolomide in this study. The reason for this might be that U251 and LN229 cells have been resistant to temozolomide.

As mentioned above, PSEN1 played an important role in tumor progression and drug resistance. Orzechowska and his colleagues found that overexpression of PSEN1 was beneficial to improving the disease-free survival of intraluminal breast cancer, while low expression of PSEN1 was instrumental in the disease-free survival of triple-negative breast cancer (24). More importantly, however, PSEN1 was negatively correlated with chemotherapy resistance of bladder cancer cells. The decrease in the PSEN1 level suppressed apoptosis induced by drug in bladder cancer cells, while overexpression of PSEN1 exacerbated drug-induced cell death, whose mechanism might be that the signal pathways related to DNA damage were activated by PSEN1 (25). In addition, when PSEN1 was overexpressed, chemotherapeutic and radiotherapeutic resistance of esophageal squamous cell carcinoma abated, thereby inducing cell apoptosis (45). In this study, we found that along with the decrease in proliferation and increase in apoptosis, the expression of PSEN1 was upregulated in U251/LN229 cells and xenograft tumor intervened by SCU and its combination with C_18_H_17_NO_6_, suggesting that this drug combination might promote glioma apoptosis and enhanced the sensitivity of glioma to the drugs themselves by upregulating PSEN1.

What signals also had changed when PSEN1 was upregulated? Firstly, by transfecting PSEN1 mutant into SH-SY5Y cells, Vestling and his colleagues observed that the cells were more inclined to die, whose mechanism might be that AKT protein was downregulated by the PSEN1 mutant (22). Moreover, in familial Alzheimer’s disease, PSEN1 mutation inhibited PI3K/AKT signal activation, leading to cell apoptosis and progression of Alzheimer’s disease (26). In cancer studies, by TargetScan and Gene Ontology analysis, Tao and his colleagues found that the target genes of differentially expressed miRNAs between aristolochic acid-induced upper urinary tract cancer and noncancer tissues were AKT3, FGFR3, PSEN1, VEGFa, and AR, which regulated cell proliferation and tumor progression in FGFR3 and AKT pathways (27). To sum up, we believed that PSEN1 might interact with the PI3K/AKT signaling pathway to play an antiglioma role.

To authenticate this hypothesis, we quantified the level of PI3K, p-PI3K, AKT, and p-AKT by Western blot. At first, we found that the protein level of PI3K, p-PI3K, AKT, and p-AKT in glioma cells was upregulated in comparison with normal astrocytes and neurons (see **Supplementary Figure S5**). The results then demonstrated that SCU and its combination with C_18_H_17_NO_6_ deactivated the PI3K/AKT signaling pathway *in vitro* and *in vivo*. Therefore, the PSEN1 might have been mutational in glioma cells. As reported previously, individual or combined use of small molecule inhibitors targeting PI3K, AKT, and mTOR, three main nodes of PI3K/AKT/mTOR signaling pathway, could achieve therapeutic effect on cancers (46). In leukemia, selective mTORC1 inhibitor RAD001 and novel allosteric AKT inhibitor MK-2206 synergistically reduced the cell viability, induced cell cycle arrest in G0/G1 phase, and led to apoptosis and autophagy in precursor acute lymphoblastic leukemia cell lines and primary cells (47). Moreover, repression in MEK and PI3K-AKT pathways could synergistically inhibited the proliferation of BaF3 cells, expressing IL7RA, JAK, and RAS mutants and was cytotoxic to primary T-cell acute lymphoblastic leukemia (48). In hepatocellular carcinoma, mTOR blockade not only hindered PI3K/AKT/mTOR signal but also reduced the concentration and stability of FBP1/2, thus restraining the proliferation of hepatocellular carcinoma cells (30). Therefore, in this study, scutellarin and C_18_H_17_NO_6_ might act as inhibitors of PI3K-AKT signaling, to suppress the proliferation and induce the apoptosis of glioma. Taken together, scutellarin and its combination with C_18_H_17_NO_6_ might upregulate PSEN1 but abated PI3K/AKT signaling in glioma.

In conclusion, scutellarin and its combination with C_18_H_17_NO_6_, with acceptable toxicity, suppressed the proliferation and migration and induced the apoptosis of glioma, which was partly associated with the repression of PSEN1/PI3K-AKT signaling axis.

## Methods

### Cell Culture

Glioma cell lines U251 and LN229 cells (ATCC) were included in this study. As previously reported (32), when the cells grew to fusion degree of 80%–90%, the old culture medium was discarded and the cells were rinsed twice with 0.01 M phosphate-buffered saline (PBS) (Servicebio, Wuhan, China). The cells were then digested with 0.25% trypsin (Hyclone, Logan, UT, USA) for 3–5 min, and the digestion was terminated with complete medium (89% Dulbecco’s modified Eagle’s medium (DMEM) with high glucose or DMEM F12 (Hyclone) + 10% fetal bovine serum (FBS; Hyclone) + 0.1 mg/ml streptomycin and 100 U/ml penicillin (Hyclone)). After centrifugation, the cells were collected and resuspended with fresh complete medium, and then seeded in cell plates.

In order to acquire images of cell morphology, the cells were inoculated in 96-well plates (Corning, Corning, NY, USA) with 3,000–5,000 cells per well and were placed in a cell culture incubator (Thermo, Waltham, MA, USA) containing 5% CO_2_, 95% humidity, and 37° for overnight. Then the drugs, including scutellarin (SCU, Kunming Longjin Pharmaceutical Co., Ltd., Kunming, China) and C_18_H_17_NO_6_ (provided by Professor Xiao-Qiong He, Kunming Medical University, Kunming, China), were added. After the drug intervention for 24, 48, or 72 h, the bright field photographs were taken using inverted fluorescence microscopy camera system (Leica, Wetzlar, Germany) and at least five fields were taken from each well.

For primary culture, astrocytes and neurons were included and used to toxicity analysis. According to the methods reported previously (32), astrocytes were isolated, cultured and purified. With the same procedures, neurons were isolated from neonatal SD rats. All the procedures of acquisition and execution of neonatal SD rats were conducted with approval by the Ethical Committee of Kunming Medical University (reference number: kmmu 2018016).Then, neurons were resuspended with complete medium and cultured in the cell plates coated with 10μg/ml poly-L-lysine at 37°C for 30 minutes. Four hours later, the complete medium was replaced by neuron-specific medium, consisting of 98% Neurobasal medium(Gibco) and 2% B27 supplement (Gibco). After that, the medium was replaced with neuron-specific medium every three days.

### Immunofluorescence

The purity of astrocytes and neurons was identified by immunofluorescence staining. The procedures were as described previously (32). Briefly, astrocytes and neurons were firstly fixed with 4% paraformaldehyde (Servicebio). After washing with 0.01 M PBS, 5% goat serum (Invitrogen, Waltham, MA, USA) and 3‰ Triton X-100 was added and incubated at 37°C for 30 min. Then, anti-GFAP primary antibody and anti-Tuj1 primary antibody (see **Table 1**) were added to astrocytes and neurons, respectively. After incubating overnight at 4°C, Cy3 goat antimouse secondary antibody (1:200, Jackson, Bar Harbor, ME, USA) was added and incubated at 37°C for 1 h. Finally, the nuclei were stained with 4′,6-diamidino-2-phenylindole (DAPI) (Beyotime). Fluorescence images were captured by inverted fluorescence microscopy camera system (Leica).

**Table 1:** The directions of all primary antibodies used in this study.

Primary antibody	Manufacturer	Attribute	Dilution
Aanti-PI3K	CST	Rabbit	1:500
Anti-p-PI3K	CST	Rabbit	1:500
Anti-AKT	CST	Rabbit	1:500
Anti-p-AKT	CST	Rabbit	1:500
Anti-β-actin	Abbkine	Mouse	1:2,000
Anti-PSEN1	CST	Rabbit	1:1,000
Anti-GFAP	Abcam	Mouse	1:200
Anti-Tuj1	Abcam	Mouse	1:400

### Cell Viability Analysis

Cell viability was detected by CCK-8 kit (DOJINDO, Japan). Briefly, the cells were seeded in 96-well plates with 3,000–5,000 cells/well. The cell plates were incubated in a cell culture incubator for 24 h, and then the drugs were added. After 48 h of intervention, 10 μl of CCK-8 reagent was added into each well and incubated at 37°C for 2 to 4 h. The absorbance (OD value) was measured by Multiskan Spectrum Microplate Spectrophotometer (Thermo) at a wavelength of 450 nm. The inhibition rate, used for subsequent data statistics and calculation of IC_50_, was calculated by the formula inhibition rate = (Ac − As)/(Ac − Ab) * 100%, in which As was absorption of drug-added wells (experimental groups), Ac was absorption of solvent-added wells (control group), and Ab was absorption of blank control wells (medium wells containing CCK-8 reagent). Three replicates were set in each group.

### Real-Time Analysis of Cell Proliferation and Migration

In order to monitor the proliferation and migration of U251 cells in real time, the experiment was carried out with xCELLigence Real Time Cell Analyzer (ACEA). The instrument could be placed in an incubator containing 5% CO_2_, 95% humidity, and at 37°C. More importantly, it could integrate the relative impedance change of microelectronic sensors on cell plate bottom, whose output was the cell index used for evaluating the cell proliferation or migration ability.

E-plate 16 (Roche Diagnostics GmbH, Basel, Switzerland) was used for proliferation assay of U251 cells. U251 cells were seeded at E-plate 16 at 5,000 cells/well. After the cells were attached overnight, the medium was changed to complete medium containing different concentrations of the drugs. From the time of cell inoculation, the cells were monitored every 15 min for a total of 4 days.

Cell migration was assessed by a specially designed CIM-plate 16 (Roche Diagnostics GmbH), in which each pore consisted of the upper and lower chambers separated by an eight-microporous membrane. This plate possessed the appearance of a traditional Transwell chamber. Culture medium containing 10% FBS was added in the lower chamber, while U251 cells, intervened by drugs for 48 h and resuspended with serum-free medium, were inoculated into the upper chamber with 40,000 cells/well. The impedance, produced by cells attached to the bottom of the upper chamber, was converted into cell index, which was monitored by CIM-plate 16 every 15 min for a total of 2 days.

Data analysis was carried out by xCELLigence Real Time Cell Analyzer software 1.2 attached to the instrument. Each group contained three independent replicates.

### Plate Clone Formation Assay

As described previously (32), on the first day of the experiment, 1,000 cells were seeded per well in six-well plates (Corning) and then the plates were placed in a 37°C incubator overnight. After the intervention drugs were added, the cells were cultured in the incubator at 37°C until the 10th day, during which the medium was changed once every 3 days and the state of the cells was observed. On the 10th day, the old medium was discarded, and the cells were washed with 0.01 M PBS for three times and then fixed with 4% paraformaldehyde for 15 min. After washing, the cells were stained with 0.5% crystal violet (Beyotime) for 10 min and rinsed three times with 0.01 M PBS. Finally, the digital camera (Nikon) was used to take a picture of the formed clones and the clones were counted. Here, three replicate wells were also set in each group.

### EdU Incorporation Assay

EdU incorporation assay were performed by EdU cell proliferation detection kit (RiboBio, Guangzhou Science City, China) according to the manufacturer’s instructions. In brief, the cells in logarithmic phase were inoculated into 96-well plates with 5,000/well. When the cells were attached to the wall overnight, they were intervened with drugs for 48 h. Then, 100 μl 50 μM EdU medium was added into each well and the 96-well plates were incubated in an incubator at 37°C for 2 h. After washing with 0.01 M PBS twice, the cells were fixed for 30 min by 4% paraformaldehyde at room temperature. The fixed fluid was then abandoned, and the remaining aldehydes were neutralized by 2 mg/ml glycine. Then the cells were washed with 0.01 M PBS for 5 min and perforated with 0.5% TritonX-100 solution for 10 min. Similarly, after rinsing with 0.01 M PBS, the cells were incubated with 1× Apollo^®^ dyeing solution for 30 min in a bleaching shaker at room temperature and in the dark. Finally, 1× Hoechst 33342 reaction solution was prepared and used to stain the nucleus for 30 min. Images were acquired immediately after staining. The proliferation rate was equal to the ratio of EdU-positive cells (red) to Hoechst-positive cells (blue), which was multiplied by 100%.

### TUNEL Assay

Firstly, 5,000 cells per well were seeded in 96-well plates. After adherence to the wall, the cells were intervened by drugs for 48 h and then stained by TUNEL detection kit (Roche). Following the instructions, the cells were fixed in 4% paraformaldehyde for 15 min at 15°C–25°C, and then sodium citrate antigen repair solution (Beyotime) containing 0.1% Triton X-100 (SolarBio, Beijing, China) was added and incubated on ice (2°C–8°C) for 2 min to permeabilize the cells and repair the antigens. The prepared 50-μl TUNEL reaction mixture, consisting of 50 μl TdT and 450 μl fluorescein-labeled dUTP solution, was then added into each well and the reaction was conducted at 37°C for 1 h in the dark. After rinsing with 0.01 M PBS, the cell nuclei were stained with 5 μg/ml DAPI (Beyotime). Ultimately, the stained samples were photographed by inverted fluorescence microscopy camera system (Leica). TUNEL and DAPI-labeled cells were counted, respectively. The apoptotic rate was equal to the ratio of TUNEL-positive cells (red) to DAPI-positive cells (blue), which was multiplied by 100%.

### Cell Cycle and Apoptosis Analysis by Flow Cytometry

Flow cytometry was also carried out as described previously (32). Cell cycle and apoptosis of the cells intervened by the drugs for 48 h was analyzed.

For cell cycle analysis, the collected cells were firstly fixed by 70% ethanol at 4°C overnight and then labelled with propidium iodide/ribonuclease staining solution (BD Bioscience, Franklin Lakes, NJ, USA) for 15 min in the dark. Afterwards, more than 10,000 cells were detected by flow cytometry according to the standards of the operating instructions.

Cell apoptosis analysis was conducted with Annexin-V cell apoptosis detection kit (BD Bioscience). At first, the intervened cells were stained with the 20ug/ml Annexin-V labeled with FITC for 30min at room temperature and in the dark. Then they were reacted with 50μg/ml PI for 5min under the same conditions. After adding 400μl of combined buffer, the samples were tested by flow cytometry immediately according to the standards. In each test, 20000 to 30000 cells were analysed by flow cytometry, in which the living cells were not marked with both Annexin-V and PI, the early apoptotic cells were stained with Annexin-V only, the late apoptotic cells were stained with both Annexin-V and PI, and the mechanically injured cells were only labelled with PI.

### Wound-Healing Assay

The lateral migration of cells was evaluated by wound-healing assay. Firstly, the cells were inoculated into six-well plates with 1 × 10^6^ cells/well and attached overnight. Then the symbol “#” was drawn on the bottom of the wells with a 10-μl pipette tip. After the exfoliated cells were removed, the fresh complete medium containing drugs were added. At 0, 12, 24, 36, and 48 h of drug intervention, the scratches were photographed with inverted fluorescence microscopy camera system (Leica). Each group acquired 10 fields, and each field was obtained from the same coordinate at all time points. The shortest distance of the scratches at 0, 12, 24, 36, and 48 h was measured by ImageJ software (NIH). The cell migration distance was equal to the difference between the shortest distance of 12, 24, 36, and 48 h and the shortest distance of 0 h, and then the migration rate was calculated as follows: migration rate = (the shortest distance at 0 h − the shortest distance at *X*h)/the shortest distance at 0 h * 100%.

### Transwell Assay

The vertical migration ability was detected by Transwell assay. This experimental device included an upper chamber (Transwell Chamber (Millipore, Burlington, MA, USA)) and a lower chamber, in which the bottom of the upper chamber was an 8-μm microporous membrane and the lower chambers were the wells of 24-well plates; 5 × 10^4^ cells intervened by drugs for 48 h were resuspend with serum-free medium and seeded into the upper chamber, while the complete medium containing 10% serum was added into the lower chamber. After 48 h of migration, the noninvasive cells in the upper chamber were gently removed with a cotton swab. However, the invasive cells on the bottom of the upper chamber were fixed with 4% paraformaldehyde for 30 min at room temperature and stained with 0.5% crystal violet for 10 min. After rinsing, the cells on the microporous membrane at the bottom of the upper chamber were photographed by a microscope. Fifteen fields were captured in each group, and the invasive cells were counted and statistically analyzed.

### RNA Extraction and Quantitative Reverse Transcription Polymerase Chain Reaction

RNA extraction and quantitative reverse transcription polymerase chain reaction (qRT-PCR) were performed as described previously (32), in which the micropipettes (Eppendorf), pipette tips (AXYGEN, Corning, NY, USA), PCR instrument (Bio-Rad, Hercules, CA, USA), Trizol lysate (TaKaRa, Kusatsu, Japan), First Strand cDNA Synthesis Kit (Thermo), and PCR Master Mix (2×) (Thermo) were needed. In this study, 19 candidate differential genes were screened out, so they were quantitatively analyzed by qRT-PCR. Additionally, the primers of all the 19 genes are shown in **Supplementary Table S1**.

### Western Blot

Referring to the previous description (32), protein extraction and immunoblotting were performed. The antibodies directed against the following proteins, including PI3K, p-PI3K, AKT, p-AKT, and β-actin were used, and their directions are shown in **Table 1**. HRP-conjugated secondary antibodies (1:5,000, GeneTex, Irvine, CA, USA) were also used. Other materials consisted of electrophoresis and membrane transfer device (Bio-Rad), RIPA lysate (Beyotime), protease inhibitor (cocktail) (Roche), ECL chemiluminescence substrate Kit (Biosharp, Hefei, China), defatted milk powder (Wondersun, Harbin, China), protein marker (Bio-Rad), BCA kit (Beyotime), and SDS-PAGE gel rapid preparation kit (Beyotime). Blotting was captured by Molecular Imager ChemiDocTM XSR+ Gel Imaging System (Bio-Rad) and analyzed using ImageJ software (NIH). In semiquantitative analysis of the target protein, each sample was normalized to β-actin.

### Tumor Xenograft Models and Therapeutic Regimens

All animal experiments were performed according to standard guidance of experimental animals provided by the Ethical Committee of Kunming Medical University. Female BALB/c nude mice (weighing approximately 20 g) between 4 and 6 weeks of age were purchased from Beijing Institutes for Biological Sciences (Beijing, China). To assess the antiglioma activity of scutellarin and C_18_H_17_NO_6_ in a xenograft model *in vivo*, the mice were randomly divided into four groups (*n* = 5): control (10% DMSO), scutellarin 50 mg/kg (SCU 50 mg/kg), C_18_H_17_NO_6_ 20 mg/kg, and SCU 50 mg/kg + C_18_H_17_NO_6_ 20 mg/kg (SCU+C_18_H_17_NO_6_). U251 cells (5 × 10^6^/mice) were subcutaneously injected into the right side of the armpits of BALB/c nude mice. Four weeks after implantation, the drug administration was initiated, and the dosing frequency was once every 2 days. Meanwhile, the tumor growth was examined. The tumor width and length and body weight of nude mice were also measured every 2 days. The tumor volume was calculated as (length × width × width)/2. For 5 weeks of therapy, the mice were killed and the tumor tissue was sampled for Western blot. Moreover, the blood was collected and centrifuged for acquiring the serum.

### Automatic Blood Biochemical Examination

As described previously (49), the Beckman Coulter Chemistry Analyzer (AU480) (Beckman Coulter K.K., Tokyo, Japan) was used to detect the serum level of the indicators that reflect the liver and kidney function. After the standard reagents (Beckman Coulter Laboratory Systems Co., Lid, Suzhou, China) was checked, ISE High Serum Standard and ISE Low Serum Standard (Beckman Coulter, Inc. Brea, CA 92821, USA) were then located in the S-H and S-L positions, respectively, for all ion calibrations. Then, the sample cups with Control Serum 1 and Control Serum 2 (Beckman Coulter Ireland Inc., Co. Clare, Ireland) were placed on the sample rack (Beckman Coulter K.K., Tokyo, Japan) for quality control tests. After these three tests were completed, the results were transmitted to the Lis 2.2 software. When all the tests were passed, blood biochemical examination of the samples could be performed. Three hundred microliters of the serum sample in clean sample cups were detected, and the items reflecting the liver and kidney function was included. The results were also displayed on the Lis 2.2 software.

### Statistical Analysis

In this study, the data were expressed as mean ± SD. One-way ANOVA was performed on continuous data from three independent groups and above, general linear model-repeated measure was used for analyzing the repeated measurement data, and analysis of variance of factorial design was for the factorial design data. All the analyses were performed using SPSS 16.0 software. As long as *p* < 0.05, the difference was statistically significant.

## Data Availability Statement

The datasets presented in this study can be found in online repositories. The names of the repository/repositories and accession number(s) can be found below: http://gdac.broadinstitute.org/runs/stddata:2016_01_28/data/GBM/20160128/, Index of/runs/stddata:2016_01_28/data/GBM/20160128.

## Ethics Statement

The animal study was reviewed and approved by the Ethical Committee of Kunming Medical University.

## Author Contributions

T-HW, R-RW, and X-QH participated in the guidance and design of the study and the revision of the paper. X-YH was responsible for the design of the study, the manuscript writing and revision, and data analysis and description and participated in all the tests. Q-JX performed qPCR test, supervised the experiments, and participated in the manuscript revision. YX, X-MZ, and SL performed cell culture, cck8 assay, and wound-healing assay and the data analysis. All authors contributed to the article and approved the submitted version.

## Funding

This study was supported by the Key Research and Development Program of Sichuan Province (No. 2020YFS0043) and Applied Basic Research Program of Sichuan Province (No. 2021YJ0459).

## Conflict of Interest

The authors declare that the research was conducted in the absence of any commercial or financial relationships that could be construed as a potential conflict of interest.

## Publisher’s Note

All claims expressed in this article are solely those of the authors and do not necessarily represent those of their affiliated organizations, or those of the publisher, the editors and the reviewers. Any product that may be evaluated in this article, or claim that may be made by its manufacturer, is not guaranteed or endorsed by the publisher.

## Supplementary Material

Supplementary Figure 1 Expression of the 19 CDGs in 166 glioma samples. This figure shows the expression of 19 CDGs in 166 glioma samples and the overall survival and disease-free survival of each patient.

Supplementary Figure 2 The mRNA level of CDGs in U251 cell after intervened by scutellarin and its combination with C18H17NO6. This figure shows the mRNA expression of 17 CDGs in U251 cell after intervened by scutellarin and its combination with C18H17NO6. *: *vs* Control (0.153% DMSO), #: SCU x *vs* SCU x+C18H17NO6 2μM, */#P < 0.05, **/##P < 0.01, ***/###P < 0.001 (n = 3).

Supplementary Figure 3 The mRNA level of CDGs in LN229 cell after intervened by scutellarin and its combination with C18H17NO6. This figure shows the mRNA expression of 18 CDGs in LN229 cell after intervened by scutellarin and its combination with C18H17NO6. *: *vs* Control (0.153% DMSO), #: SCU x *vs* SCU x+C18H17NO6 2μM, */#P < 0.05, **/##P < 0.01, ***/###P < 0.001 (n = 3).

Supplementary Figure 4 The genes with the same change trend in mRNA expression in U251 and LN229 cells after intervention with scutellarin alone, C_18_H_17_NO_6_ alone and the combination of scutellarin and C_18_H_17_NO_6_. **(A)** after the intervention of scutellarin alone and C_18_H_17_NO_6_ alone, the genes with the same change trend of mRNA expression in U251 and LN229 cells; **(B)** after the intervention of the combination of scutellarin and C_18_H_17_NO_6_, the genes with the same trend of mRNA expression in both cells; **(C)** after intervention with scutellarin alone, C_18_H_17_NO_6_ alone and the combination of scutellarin with C_18_H_17_NO_6_, the genes with the same change trend of mRNA expression in both cells. Note: “S” is scutellarin (SCU), “C” is C_18_H_17_NO_6_, “S+C” is the combination of SCU with C18H17NO6.

Supplementary Figure 5 The protein expression of PI3K, p-PI3K, AKT and p-AKT in normal astrocyte, neuron, U251 and LN229 cells. **(A)** Protein bands showed the change in the protein expression of PI3K, p-PI3K, AKT and p-AKT in astrocyte, neuron, U251 and LN229 cells; **(B)** Quantification of the protein expression of PI3K, p-PI3K, AKT and p-AKT in astrocyte, neuron, U251 and LN229 cells. *: *vs* Astrocyte or Neuron, *P < 0.05 (n=3).
