# Corrigendum: Combined Scutellarin and C_18_H_17_NO_6_ Imperils the Survival of Glioma: Partly Associated With the Repression of PSEN1/PI3K-AKT Signaling Axis

**DOI:** 10.3389/fonc.2022.872110

**Published:** 2022-03-07

**Authors:** Xiu-Ying He, Yang Xu, Qing-Jie Xia, Xiao-Ming Zhao, Shan Li, Xiao-Qiong He, Ru-Rong Wang, Ting-Hua Wang

**Affiliations:** ^1^Institute of Neurological Disease, Department of Anesthesiology, Translational Neuroscience Center, West China Hospital, Sichuan University, Chengdu, China; ^2^Institute of Neuroscience, Laboratory Zoology Department, Kunming Medical University, Kunming, China; ^3^School of Public Health, Kunming Medical University, Kunming, China

**Keywords:** C_18_H_17_NO_6_, PSEN1, glioma, PI3K-Akt signaling, scutellarin

The shared equal contribution made by authors Xiu-Ying He, and Yang Xu was not depicted in the article. These authors have contributed equally to this work.

The authors apologize for these errors and state that this does not change the scientific conclusions of the article in any way. The original article has been updated.

## Publisher’s Note

All claims expressed in this article are solely those of the authors and do not necessarily represent those of their affiliated organizations, or those of the publisher, the editors and the reviewers. Any product that may be evaluated in this article, or claim that may be made by its manufacturer, is not guaranteed or endorsed by the publisher.

