# Supplementary material for: Combined Scutellarin and C18H17NO6 Imperils the Survival of Glioma: Partly Associated With the Repression of PSEN1/PI3K-AKT Signaling Axis
Source: Front Oncol. 2021 Sep 9;11:663262. doi: 10.3389/fonc.2021.663262 (PMC8460401; doi:10.3389/fonc.2021.663262)
Supplement: Supplementary file 1 [file Presentation_1.pdf]

Supplementary Material

Supplementary Methods

1. Identification and annotation of the candidate differential genes (CDGs)

Differential genes were obtained from the RNA sequencing data of the TCGA database (The Cancer Genome Atlas) using Cbioportal online tool (https://www.cbioportal.org). The parameters selected for the analysis were: “Glioblastoma Multiforme (TCGA, Provisional), 604 samples included”, mRNA Expression: mRNA Expression z-Scores (RNA Seq V2 RSEM) with a z-score threshold ±2, and the entered genes consisted of all genes that selected from recently mutated genes (MutSig) and selected genes from recurrent CNAs (Gistic). Then, 19 candidate differential genes (CDGs) were screened by reviewing the study field of them. At the same time, the annotation of the 19 CDGs were acquired through GeneCards database (https://www.genecards.org) and BioGPS database (http://biogps.org/#goto=welcome).

2. The primers and qRT-PCR of the 19 CDGs

According to the gene sequence published by GenBank, the primers were designed by Primer 5.0 software and synthesized by Takara Biology Ltd. The sequences of 19 CDGs primers were shown Supplementary Table 1. For details of qRT-PCR method, see in the manuscript.

Supplementary Table 1. The primers of the 19 candidate genes

Primers	Sequence (5' to 3')
MED16 F:	GACAGCATGTCCCTGCTCTT
MED16 R:	CTGGGCAGCAGGCAGCATT
POLRMT F:	CTGGACTCCTCCCACATGAT
POLRMT R:	CAGGCCCTTCCTGTAGCAGT
PPP2R5CF:	GAGTGTCTACCATCCCCAG
PPP2R5CR:	GACTGTGAGTCTTTGGCCAG
PPP2R5E F:	CGTCATCCTTCCCATCATGTT
PPP2R5E R:	CACGCTGACGATCTGACTTGT
PSEN1 F:	CATCTTGGCTGTGATTTCAGT
PSEN1R:	CTGAGCTGTTTCAACCAGCAT
SGMS1F:	GAATGCTATGCCAGAATACT
SGMS1R:	GAAATCCTCTTGGGTTAGGT
VPS36F:	GAGTTTTACAGGCGTTTATCAG

VPS36R:	TCTTCCTGGCTGGGGTCCT
WDR20F:	CCTCAACGAGTTTGCTTTCT
WDR20R:	CAGCTCCACTGAGTCAAAGT
CCNK F:	CCTCACAACTTGAAGGACT
CCNK R:	CCCAAACGTGTGCCCACAT
DCAF5F:	CTGCTCACTCAGGACTTTCAG
DCAF5R:	GCATTGACACAGCCGAAGTG
DLST F:	CCTCTAGGGAGACGTTCCCT
DLST R:	CACCTGACATCTCCCTCTGT
FAF1F:	GTCTTTATGTCCTTACACCAG
FAF1R:	GGTGATGATCAGCATGAAGT
HBS1L F:	CTCAACTTAGTGGTCATTGGT
HBS1L R:	CCCAGAAGATAAAGCATATGG
KPNA3F:	GAACTTCTGCACAGACTCAAG
KPNA3R:	CTACATTGAGGACCATCACCT
FAM133B F:	GGTCCAATCCAGTCTTCAG
FAM133B R:	TCAGCCAAAGCCTTGGAG
MARK3F:	CTCAGTTGAATCCAACAAGT
MARK3R:	CCTCCACTTGCATATTCCAT
CUL4A F:	CTGCAGCACTGGAGCGAGT
CUL4A R:	CAGGTCTTGGACCATGTCT
TSPAN31F:	GTGGGAGTCTTCCTTCTCCT
TSPAN31R:	GCCAGACATGAGCAAGAGAT
CDC40F:	GAACATTTATTGGTCACAGT
CDC40R:	CTTGATATACACTGTCCTGT

Note: F, Forward, forward primer; R, Reverse, reverse primer.

3. Statistics and plotting

For the details of the statistics and plotting, see the "**Methods**" in the manuscript. In addition, Veen diagram was implemented by Venny 2.1.0 online software (http://bioinfogp.cnb.csic.es/tools/venny/index.html) and Jvenn online tool (http://jvenn.toulouse.inra.fr/app/example.html).

Supplementary Result

1. 19 genes that vary by 15% or more in glioma population were identified as CDGs

The mRNA expression of certain specific genes in glioma, selected from recently mutated genes (MutSig) and recurrent copy number alterations (Gistic), was explored by analyzing RNA sequencing data from 166 samples of 160 glioma patients in the TCGA database. Of the 166 samples, 39 genes were changed in samples of 15% or more (Supplementary Table 2), which all were differentially expressed genes with two-fold and more difference in expression between normal tissues and glioma tissues (Supplementary Table 2). After reviewing the study field of the 39 genes, a total of 19 were identified as candidate differential genes (CDGs), which all were associated with tumor characteristics and studied in tumor field, but were rarely reported in glioma studies (Supplementary Figure 1). Supplementary Figure 1 showed the expression of the 19 CDGs in the 166 samples and the overall survival and disease-free survival of each patient.

In addition, these 19 CDGs were annotated by the GeneCards database and the BioGPS database. It was found that among these 19 genes, SGMS1, FAF1 and PSEN1 were involved in apoptosis, PPP2R5C, MARK3 and TSPAN31 were associated with cell proliferation, CCNK and CUL4A were related to cell cycle regulation, DCAF5, WDR20, VPS36, HBS1L, KPNA3, FAM133B and CDC40 were involved in transcriptional and post-transcriptional regulation, VPS36, HBS1L and PPP2R5E were linked with signal transduction, and DLST, POLRMT and MED16 were relevant to energy metabolism (Supplementary Table 3), whose biological progresses were also correlated with the development of tumors. Therefore, the mRNA expression of the 19 CDGs in U251 and LN229 cells intervened by SCU and its combination with $C_{18}H_{17}NO_6$ 2μM was detected by RT-qPCR in the next.

Supplementary Table 2. 39 genes with 15% or more changes in glioma population

GENE_SYMBOL	NUM_CASES_ALTERED	PERCENT_CASES_ALTERED
EGFR: EXP>=2 EXP<=-2;	79	48%
PDGFRA: EXP>=2 EXP<=-2;	33	20%
KEL: EXP>=2 EXP<=-2;	26	16%
SGMS1: EXP>=2 EXP<=-2;	56	34%
KLHL9: EXP>=2 EXP<=-2;	50	30%
DLST: EXP>=2 EXP<=-2;	47	28%
DCAF5: EXP>=2 EXP<=-2;	39	23%

MTAP: EXP>=2 EXP<=-2;	37	22%
WDR20: EXP>=2 EXP<=-2;	37	22%
POLRMT: EXP>=2 EXP<=-2;	35	21%
PDGFRA: EXP>=2 EXP<=-2;	33	20%
VPS36: EXP>=2 EXP<=-2;	33	20%
HBS1L: EXP>=2 EXP<=-2;	34	20%
FAF1: EXP>=2 EXP<=-2;	32	19%
AKT1: EXP>=2 EXP<=-2;	32	19%
CCNK: EXP>=2 EXP<=-2;	32	19%
EXD2: EXP>=2 EXP<=-2;	32	19%
MED16: EXP>=2 EXP<=-2;	32	19%
AGAP2: EXP>=2 EXP<=-2;	30	18%
PPP2R5C: EXP>=2 EXP<=-2;	30	18%
PPP2R5E: EXP>=2 EXP<=-2;	30	18%
IDI2: EXP>=2 EXP<=-2;	30	18%
PSEN1: EXP>=2 EXP<=-2;	28	17%
FCF1: EXP>=2 EXP<=-2;	29	17%
ZC3H14: EXP>=2 EXP<=-2;	28	17%
RNF146: EXP>=2 EXP<=-2;	28	17%
TMEM259: EXP>=2 EXP<=-2;	29	17%
KPNA3: EXP>=2 EXP<=-2;	26	16%
FAM133B: EXP>=2 EXP<=-2;	26	16%
MARK3: EXP>=2 EXP<=-2;	26	16%
MAX: EXP>=2 EXP<=-2;	27	16%
FIG4: EXP>=2 EXP<=-2;	26	16%
VTA1: EXP>=2 EXP<=-2;	26	16%
REPS1: EXP>=2 EXP<=-2;	26	16%
C19ORF12: EXP>=2 EXP<=-2;	27	16%
CUL4A: EXP>=2 EXP<=-2;	26	16%
IST1: EXP>=2 EXP<=-2;	27	16%
TSPAN31: EXP>=2 EXP<=-2;	25	15%

CDC40: EXP>=2 EXP<=-2;	25	15%

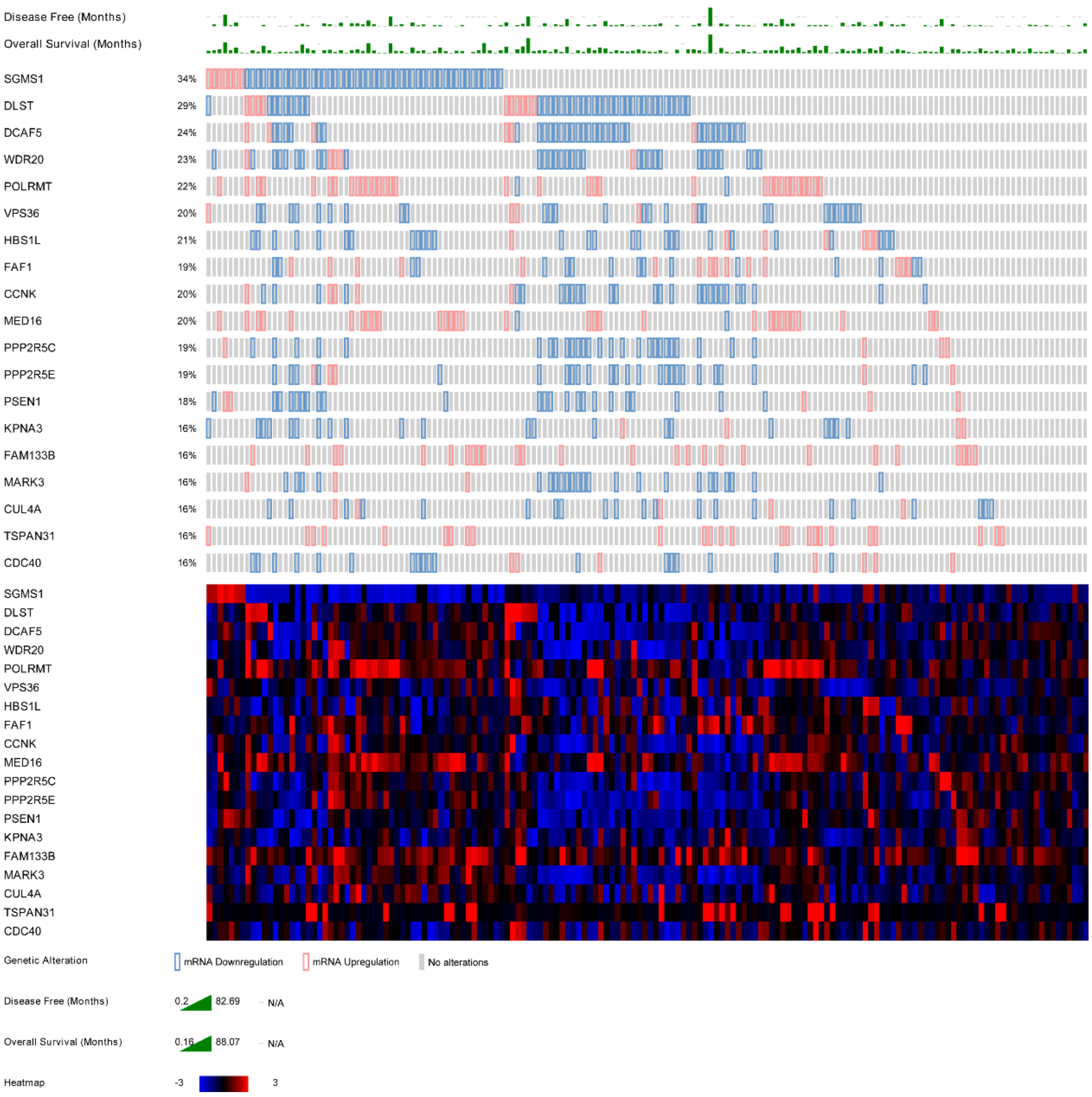

Supplementary Figure 1. Expression of the 19 CDGs in 166 glioma samples

This figure shows the expression of 19 CDGs in 166 glioma samples and the overall survival and disease-free survival of each patient.

2. The mRNA expression of 19 CDGs in glioma cells after the intervention of scutellarin and its combination with $C_{18}H_{17}NO_6$

After intervention by scutellarin and its combination with $C_{18}H_{17}NO_6$ for 48h, the genes with significant mRNA changes in U251 cell were POLRMT, PSEN1, VPS36, TSPAN31, PPP2R5C, SGMS1, DLST, HBS1L, KPNA3 and FAM133B (Supplementary Figure 2), in LN229 cell were MED16, POLRMT, PPP2R5E, PSEN1, SGMS1, VPS36, WDR20, DCAF5, TSPAN31, CCNK,

DLST, FAF1, HBS1L, FAM133B, MARK3 and CUL4A (Supplementary Figure 3), and in both cells were POLRMT, PSEN1, VPS36, SGMS1, TSPAN31, DLST, HBS1L and FAM133B (Supplementary Figure 2 and 3). Furthermore, the change trend of the mRNA expression of the 19 CDGs in both cells intervened by SCU alone, $C_{18}H_{17}NO_6$ alone, and the *combination* was intersected. As shown in Supplementary Figure 4, after intervened by the single-agent SCU and the single-agent $C_{18}H_{17}NO_6$, the genes with the same trend in mRNA expression in both cells were MED16, POLRMT, PSEN1, VPS36, MARK3, CUL4A, PPP2R5E, WDR20, HBSL1 and FAM133B, in which except for the decrease in PPP2R5E mRNA expression, all others were elevated (Supplementary Figure 4A). After SCU was combined with $C_{18}H_{17}NO_6$, the genes with the same change trend in mRNA expression were POLRMT, PSEN1, VPS36, MARK3, CUL4A and WDR20 in both cells, which were all elevated (Supplementary Figure 4B). However, after intervened by SCU alone, $C_{18}H_{17}NO_6$ alone, and the combination of SCU and $C_{18}H_{17}NO_6$, the genes with the same change trend in mRNA were POLRMT, PSEN1, VPS36, MARK3, CUL4A and WDR20 in both cells, which were all upregulated (Supplementary Figure 4C), while only POLRMT, PSEN1 and VPS36 mRNA was changed significantly by SCU and its combination with $C_{18}H_{17}NO_6$ (Supplementary Figure 2 and Supplementary Figure 3). Of the 3 genes, PSEN1 was involved in apoptosis, VPS36 was related to transcriptional regulation and endocytosis transport, and POLRMT was associated with mitochondrial gene expression (Supplementary Table 3). Since SCU and its combination with $C_{18}H_{17}NO_6$ suppressed the proliferation of glioma cells and induced their apoptosis, we only focused on the change of PSEN1 in this study (See **Results** in the manuscript for details).

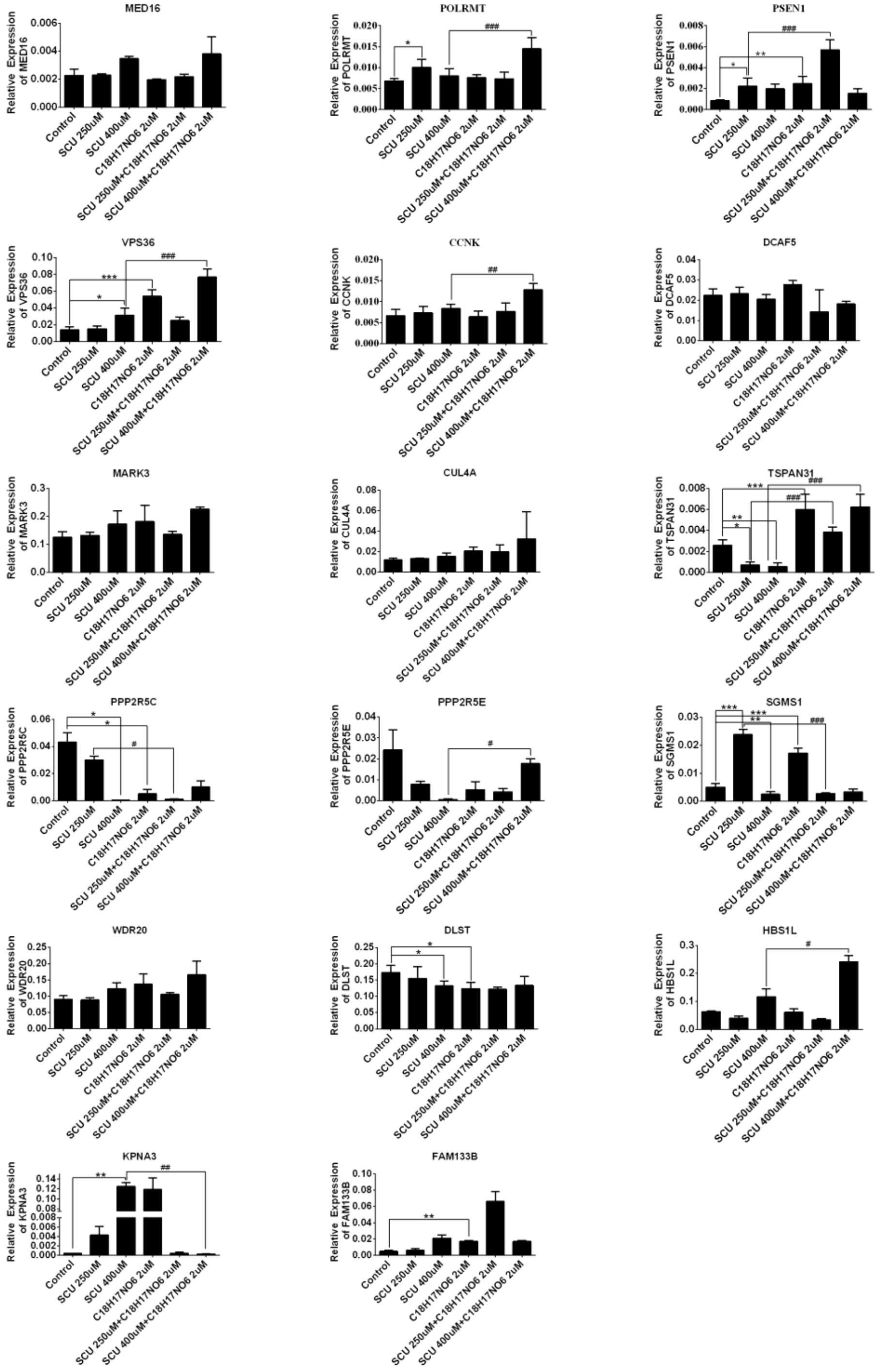

Supplementary Figure 2. The mRNA level of CDGs in U251 cell after intervened by scutellarin and its combination with $C_{18}H_{17}NO_6$

This figure shows the mRNA expression of 17 CDGs in U251 cell after intervened by scutellarin and its combination with $C_{18}H_{17}NO_6$. *: vs Control (0.153% DMSO), #: SCU x vs SCU x+C18H17NO6 2μM, */# $P < 0.05$, **/## $P < 0.01$, ***/### $P < 0.001$ ($n = 3$).

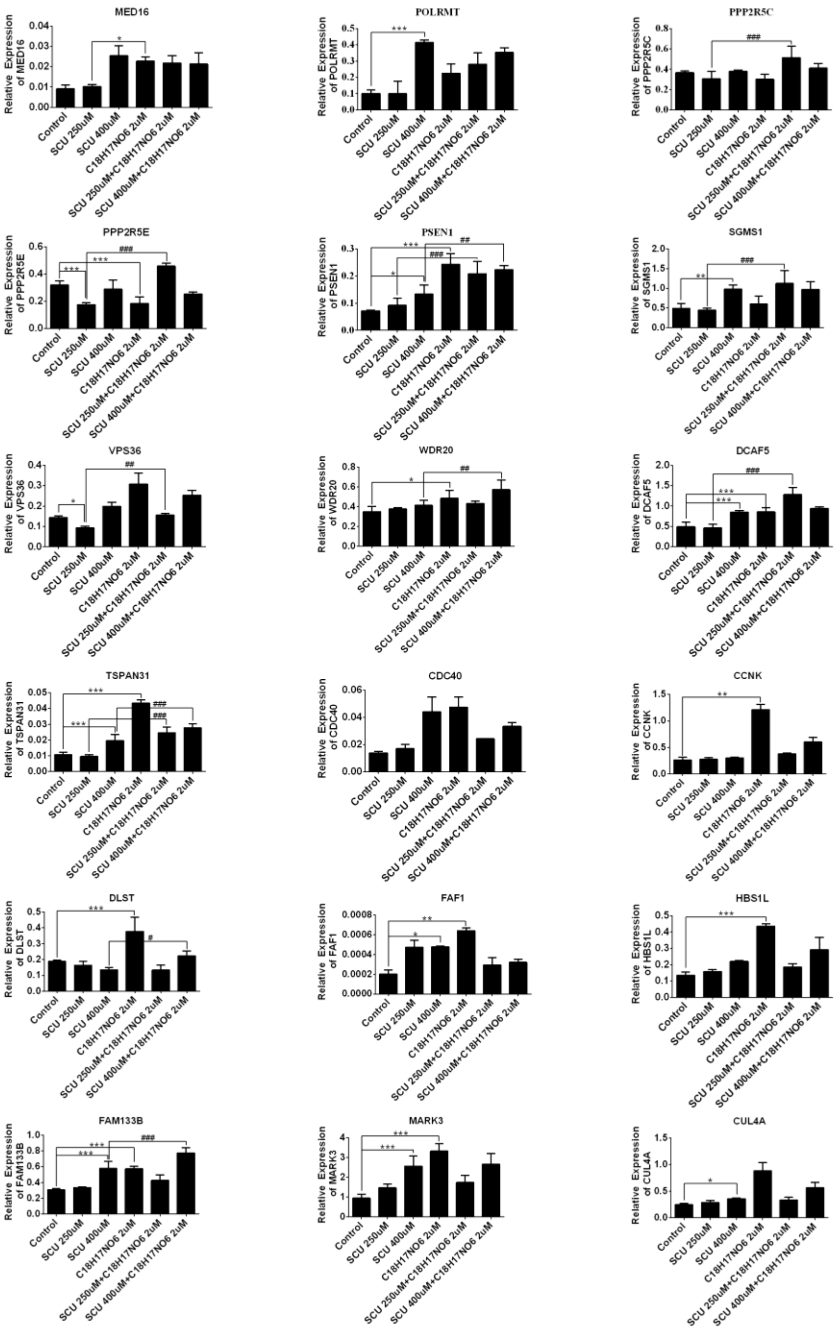

Supplementary Figure 3. The mRNA level of CDGs in LN229 cell after intervened by scutellarin and its combination with $C_{18}H_{17}NO_6$

This figure shows the mRNA expression of 18 CDGs in LN229 cell after intervened by scutellarin and its combination with $C_{18}H_{17}NO_6$. *: vs Control (0.153% DMSO), #: SCU x vs SCU x+C18H17NO6 2μM, */# $P < 0.05$, **/## $P < 0.01$, ***/### $P < 0.001$ (n = 3).

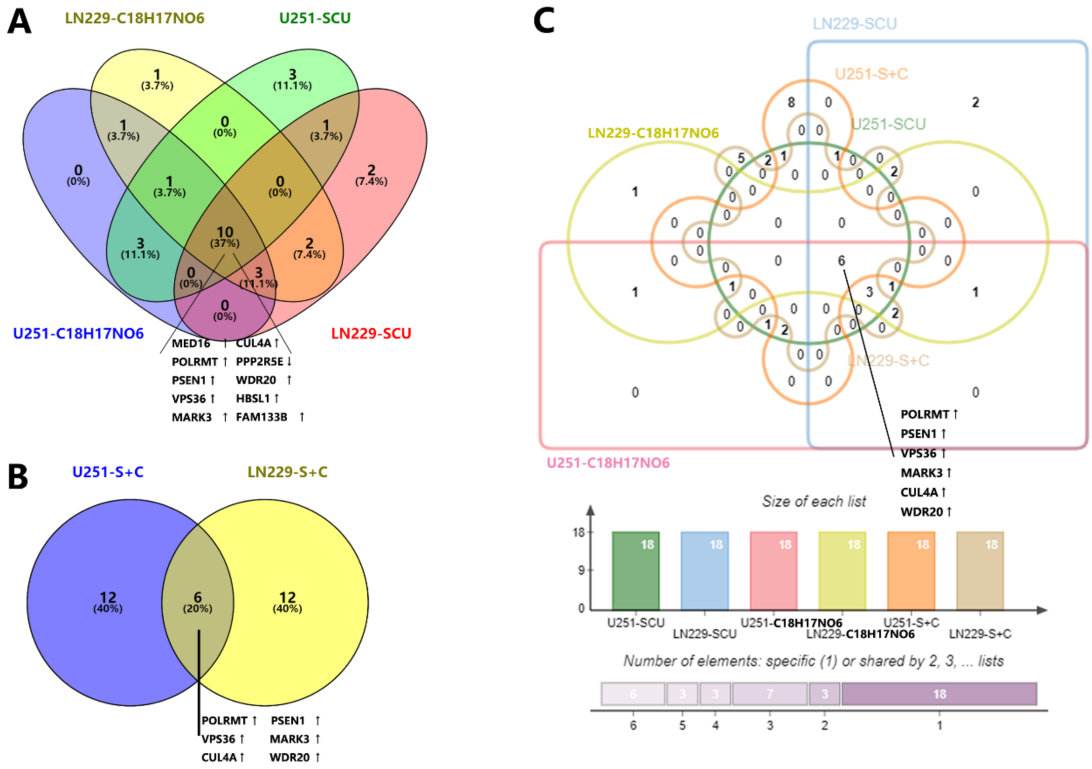

Supplementary Figure 4. The genes with the same change trend in mRNA expression in U251 and LN229 cells after intervention with scutellarin alone, $C_{18}H_{17}NO_6$ alone and the combination of scutellarin and $C_{18}H_{17}NO_6$

A. after the intervention of scutellarin alone and $C_{18}H_{17}NO_6$ alone, the genes with the same change trend of mRNA expression in U251 and LN229 cells; B. after the intervention of the combination of scutellarin and $C_{18}H_{17}NO_6$, the genes with the same trend of mRNA expression in both cells; C. after intervention with scutellarin alone, $C_{18}H_{17}NO_6$ alone and the combination of scutellarin with $C_{18}H_{17}NO_6$, the genes with the same change trend of mRNA expression in both cells. Note: “S” is scutellarin (SCU), “C” is $C_{18}H_{17}NO_6$, “S+C” is the combination of SCU with $C_{18}H_{17}NO_6$.

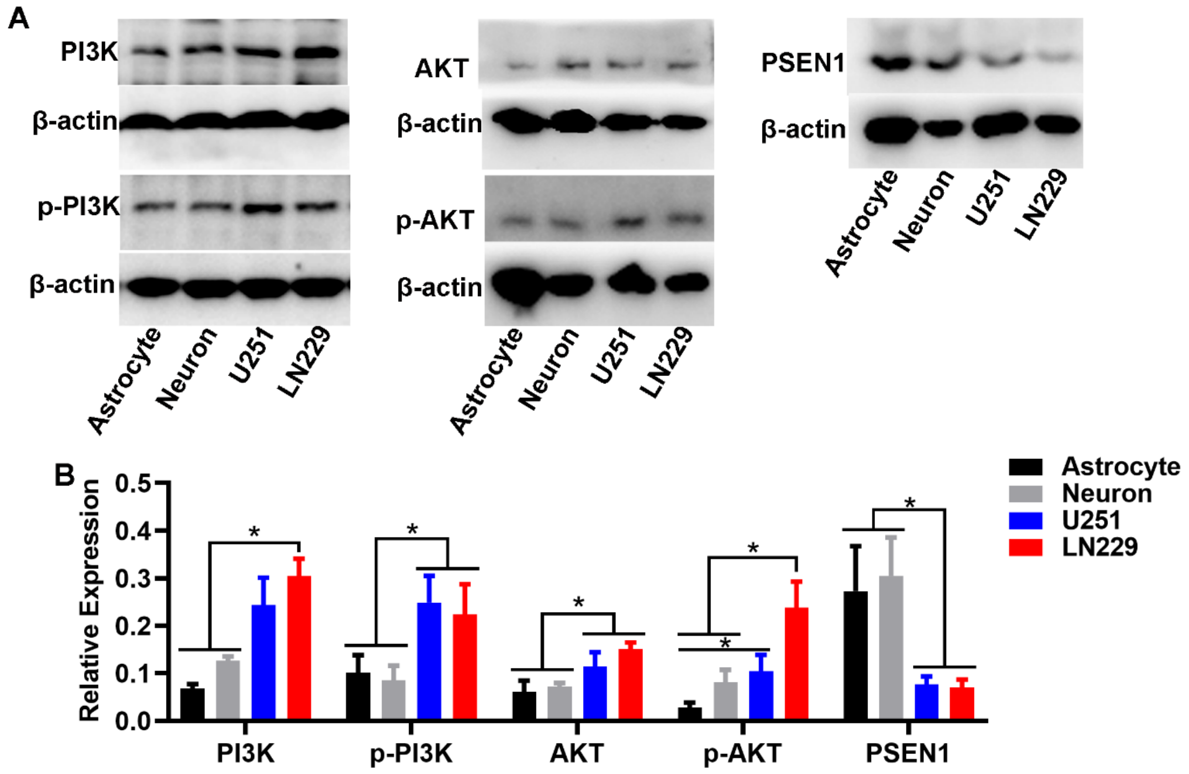

Supplementary Figure 5. The protein expression of PI3K, p-PI3K, AKT and p-AKT in normal astrocyte, neuron, U251 and LN229 cells.

A: Protein bands showed the change in the protein expression of PI3K, p-PI3K, AKT and p-AKT in astrocyte, neuron, U251 and LN229 cells; B: Quantification of the protein expression of PI3K, p-PI3K, AKT and p-AKT in astrocyte, neuron, U251 and LN229 cells. *: vs Astrocyte or Neuron, * P < 0.05 (n=3).
